# Long-Term Effects of Whole-Body Vibration on Human Gait: A Systematic Review and Meta-Analysis

**DOI:** 10.3389/fneur.2019.00627

**Published:** 2019-06-19

**Authors:** Matthieu Fischer, Thomas Vialleron, Guillaume Laffaye, Paul Fourcade, Tarek Hussein, Laurence Chèze, Paul-André Deleu, Jean-Louis Honeine, Eric Yiou, Arnaud Delafontaine

**Affiliations:** ^1^CIAMS, Université Paris-Sud, Université Paris-Saclay, Orsay, France; ^2^CIAMS, Université d'Orléans, Orléans, France; ^3^ENKRE, Saint-Maurice, France; ^4^LBMC, Université de Lyon, Lyon, France; ^5^VEDECOM, Versailles, France

**Keywords:** whole-body vibration, long-term effects, gait, biomechanics, randomized controlled trials, meta-analysis

## Abstract

**Background:** Whole-body vibration is commonly used in physical medicine and neuro-rehabilitation as a clinical prevention and rehabilitation tool. The goal of this systematic review is to assess the long-term effects of whole-body vibration training on gait in different populations of patients.

**Methods:** We conducted a literature search in PubMed, Science Direct, Springer, Sage and in study references for articles published prior to 7 December 2018. We used the keywords “vibration,” “gait” and “walk” in combination with their Medical Subject Headings (MeSH) terms. The Preferred Reporting Items for Systematic Reviews and Meta-Analyses (PRISMA) methodology was used. Only randomized controlled trials (RCT) published in English peer-reviewed journals were included. All patient categories were selected. The duration of Whole-Body Vibration (WBV) training had to be at least 4 weeks. The outcomes accepted could be clinical or biomechanical analysis. The selection procedure was conducted by two rehabilitation experts and disagreements were resolved by a third expert. Descriptive data regarding subjects, interventions, types of vibration, training parameters and main results on gait variables were collected and summarized in a descriptive table. The quality of selected studies was assessed using the PEDro scale. Statistical analysis was conducted to evaluate intergroup differences and changes after the WBV intervention compared to the pre-intervention status. The level of evidence was determined based on the results of meta-analysis (effect size), statistical heterogeneity (*I*^2^) and methodological quality (PEDro scale).

**Results:** A total of 859 studies were initially identified through databases with 46 articles meeting all of the inclusion criteria and thus selected for qualitative assessment. Twenty-five studies were included in meta-analysis for quantitative synthesis. In elderly subjects, small but significant improvements in the TUG test (SMD = −0.18; 95% CI: −0.32, −0.04) and the 10MWT (SMD = −0.28; 95% CI: −0.56, −0.01) were found in the WBV groups with a strong level of evidence (*I*^2^ = 7%, *p* = 0.38 and *I*^2^ = 22%, *p* = 0.28, respectively; PEDro scores ≥5/10). However, WBV failed to improve the 6MWT (SMD = 0.37; 95% CI: −0.03, 0.78) and the Tinetti gait scores (SMD = 0.04; 95% CI: −0.23, 0.31) in older adults. In stroke patients, significant improvement in the 6MWT (SMD = 0.33; 95% CI: 0.06, 0.59) was found after WBV interventions, with a strong level of evidence (*I*^2^ = 0%, *p* = 0.58; PEDro score ≥5/10). On the other hand, there was no significant change in the TUG test despite a tendency toward improvement (SMD = −0.29; 95% CI: −0.60, 0.01). Results were inconsistent in COPD patients (*I*^2^ = 66%, *p* = 0.03), leading to a conflicting level of evidence despite a significant improvement with a large effect size (SMD = 0.92; 95% CI: 0.32, 1.51) after WBV treatment. Similarly, the heterogeneous results in the TUG test (*I*^2^ = 97%, *p* < 0.00001) in patients with knee osteoarthrosis make it impossible to draw a conclusion. Still, adding WBV treatment was effective in significantly improving the 6 MWT (SMD = 1.28; 95% CI: 0.57, 1.99), with a strong level of evidence (*I*^2^ = 64%, *p* = 0.06; PEDro score ≥5/10). As in stroke, WBV failed to improve the results of the TUG test in multiple sclerosis patients (SMD = −0.11; 95% CI: −0.64, 0.43). Other outcomes presented moderate or even limited levels of evidence due to the lack of data in some studies or because only one RCT was identified in the review.

**Conclusions:** WBV training can be effective for improving balance and gait speed in the elderly. The intervention is also effective in improving walking performance following stroke and in patients with knee osteoarthrosis. However, no effect was found on gait quality in the elderly or on balance in stroke and multiple sclerosis patients. The results are too heterogenous in COPD to conclude on the effect of the treatment. The results must be taken with caution due to the lack of data in some studies and the methodological heterogeneity in the interventions. Further research is needed to explore the possibility of establishing a standardized protocol targeting gait ability in a wide range of populations.

## Highlights

- WBV is currently used in locomotor rehabilitation.
- WBV presents strong evidence for improving performance in the timed-up-and-go test in the elderly, but not in stroke or multiple sclerosis patients.
- WBV presents strong evidence for improving performance in the 10-meter walk test for elderly, in the 6-min walk test for stroke and knee OA patients but results are conflicting in COPD patients.
- Other outcomes present moderate or limited levels of evidence due to the lack of data or because only one RCT was identified in other pathologies.

## Introduction

Whole-body vibration (WBV) is a therapeutic method that exposes the entire body to mechanical oscillations while the patient stands or sits on a vibrating platform. This method was first used in the late nineteenth century by Charcot to treat gait disorders in neurological patients, especially in patients with Parkinson's disease (1). It is now commonly used in the physical medicine/neuro-rehabilitation fields as a prevention and rehabilitation tool for sarcopenia (2), osteoporosis (3), chronic low back pain (4), and fibromyalgia (5), among other conditions. WBV is also used in rehabilitation to improve muscle function (strength, power, and endurance) (6), muscle soreness (7), joint stability (8) and to reduce the risk of falling (9).

Several spinal and supraspinal mechanisms have been proposed to explain increased muscle activity during exposure to WBV. While there is currently no consensus, the most frequently cited mechanism is a reflex muscular contraction called tonic vibration reflex (TVR). This phenomenon has been shown to occur during direct and indirect vibratory musculo-tendinous stimulations that excite muscle spindles and enhance activation of Ia afferents, resulting in a higher recruitment of motor units and gradual development of muscle activity (10). In addition to these spinal reflexes, neuromuscular changes (11, 12), increased intramuscular temperature (10) and peripheral blood flow (13) may contribute at different levels to the increased muscular performance observed after WBV.

A recent review (14) reported a beneficial effect of long-term WBV training on balance control under static postural conditions. Since the literature appears to suggest a neuroanatomic (15) and a biomechanical continuum between standing posture and gait (16–18), Rogan et al. suggested that this beneficial effect could be extended to dynamic motor tasks such as gait (14). Such a continuum has been analyzed in stroke patients (19), for example. The most recent literature review focusing on the effect of WBV on gait, however, provided only mitigated support for this assumption (20). Based on the screening of 10 randomized controlled trials (RCT), Lindberg and Carlsson concluded there was low-quality evidence for the beneficial use of long-term WBV on gait, and acknowledged there were major limitations (20), the most important being that only one of the authors reviewed the literature. Thus, no group discussions were conducted with experts to resolve possible disagreements and reach a mutual consensus. In addition, the low number of RCT included (*n* = 10) and the absence of meta-analysis may have limited the relevance of Lindberg and Carlsson's review. Since that review was published, WBV training has been used increasingly in physiotherapy to prevent and/or treat gait disorders. Consequently, more and more experimental studies have been conducted in this area with both healthy and pathological participants.

Hence, the aim of this article is to provide an up-to-date literature review of RCT studies on the effects of long-term WBV training on gait in both healthy subjects and pathological patients. It will contribute to provide evidence-based practice for a promising non-pharmacological rehabilitative method that is both safe and cheap, and that can be used by patients at home as part of an auto-rehabilitation program.

## Materials and Methods

### Design and Literature Screening

**Figure 1:**
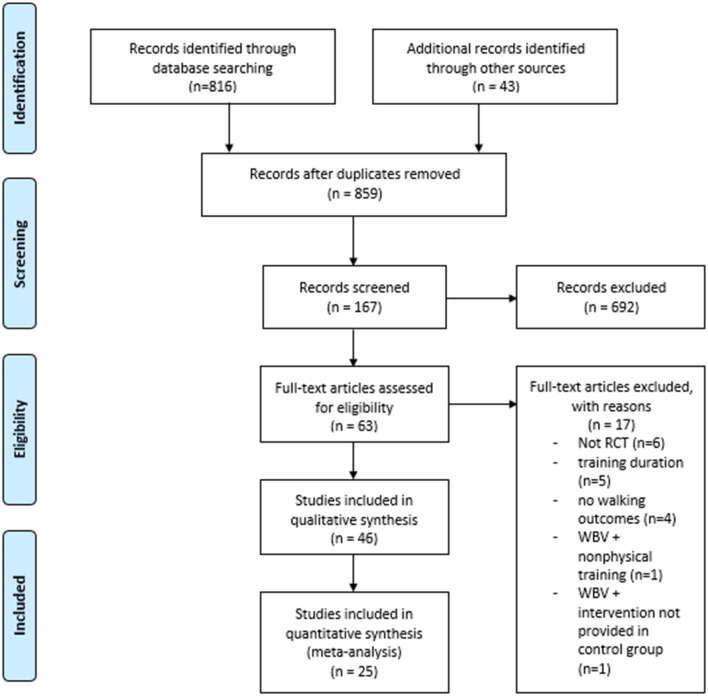
PRISMA flow chart of study selection process.

The Preferred Reporting Items for Systematic Reviews and Meta-Analyses (PRISMA) methodology was employed in this systematic review (Figure 1).

The PubMed, Science Direct, Springer and Sage databases were used for a comprehensive systematic literature search for articles published prior to 7 December 2018 with no time limit. The keywords used were: “vibration” AND (gait OR walk). More specifically, the search details specified in PubMed were: (“vibration”[MeSH Terms] OR “vibration”[All Fields]) AND ((“gait”[MeSH Terms] OR “gait”[All Fields]) OR (“walking”[MeSH Terms] OR “walking”[All Fields] OR “walk”[All Fields])).

The selection procedure was conducted by two experts in rehabilitation. Disagreements were discussed with a third expert in a group until a mutual consensus was reached. First, a review was performed on all available titles obtained from the literature search with the selected keywords. All relevant or potentially relevant titles were included in the subsequent phase. Then, the abstracts were reviewed with all relevant or potential articles included in the following phase. Finally, full-text articles were reviewed to ensure that only relevant studies were included. In the same way, reference lists of all included articles were reviewed to possibly include articles through cross-referencing.

### Inclusion and Exclusion Criteria

To be included, the studies had to meet all of the following inclusion criteria: all patient categories were selected if: gait ability was measured before and after at least 4 weeks of WBV training performed on a vibration platform; the results were based on biomechanical analyses or were clinically relevant; the control group had no intervention or performed the same physical rehabilitation, resistance, balance or endurance training as the intervention group. In addition, only RCT, articles in English, and articles published in peer-reviewed journals were included. Studies were excluded if they measured only short-term effects (<4 weeks) and if WBV was combined with non-physical training or with any intervention not provided to the control group (i.e., not only WBV effects are measured).

### Data Extraction and Main Measurements Examined

Data were extracted from the selected articles by one of the authors. The extracted data were checked by another author and disagreements were resolved with a third.

The following data were extracted for each selected article: (1) the names of the authors and the date of publication; (2) the number of subjects involved in the experiment with their characteristics and breakdown in each group; (3) WBV training details (in the following order: name of the WBV device, duration of the intervention, number of sessions, types of exercises, number of vibration sets, exposure duration per set, rest period between sets, frequency, amplitude and type of vibration) and control group details; and (4) the main outcomes related to gait with the main results (e.g., timed up-and-go test, 6-min walk test, walking speed, etc). When information could not be provided, it was indicated by a “?”.

### Quality and Risk of Bias Assessment

The PEDro scale was used to assess the risk of bias, and thus the methodological quality of the selected studies (21). The scale was chosen for its ability to provide an overview of the external (criterion 1), internal (criteria 2–9) and statistical (criteria 9 and 10) validity of RCT. The scale is divided in 11 criteria, but the first criterion is not calculated in the total score. The output of each criterion could be either “yes” (y), “no” (n) or “do not know” (?). A “y” was given a score of one point, while a “n” or “?” was assigned zero points. Studies with a total score of 5–10/10 (≥ 50%) were considered to be of high quality, and scores of 0–4/10 (<50%) as low quality (20). Two evaluators assessed the quality of the included studies independently. In the event of disagreements, a group discussion was held with a third expert to reach a mutual consensus.

### Statistical Analysis

To estimate the effect of WBV training on human gait, a meta-analysis compared the intervention groups with the control groups. Within group comparisons were added (i.e., pre vs. post intervention) when the groups were not comparable (e.g., statistical difference in outcomes at baseline or additional training in control group not provided in the intervention group). Estimations were calculated using the methodology described by Wan et al. (22) when mean and standard deviations were not reported by the authors and medians and interquartile ranges were used. The authors were contacted to request additional data when an estimation was not possible. If no response was received, the variables were excluded from meta-analysis.

Statistical analysis and figures (i.e., forest plot to facilitate the visualization of values) were produced using a random-effect model in Review Manager software (RevMan, v 5.3, Cochrane Collaboration, Oxford UK) (23). A random-effect model was used to take into account the heterogeneity between the study effects. The effect size of the interventions was reported by standard mean difference (SMD) and their respective 95% Confidence Interval (CI). In this way, the magnitude of the overall effect can be quantified as very small (<0.2) small (0.2–0.49), moderate (0.5–0.79) or large (≥0.8) (24, 25). Statistical heterogeneity was calculated using the *I*^2^ and Cochrane Q statistic tests (25). Statistical significance was set at *p* < 0.05.

### Level of Evidence

The strength of evidence of primary outcomes was established as described by Van Tulder et al. (26) based on the results of meta-analysis (effect size), statistical heterogeneity (*I*^2^) and risk of bias (PEDro scale). The level of evidence was considered strong with multiple high-quality RCT (at least two studies with a PEDro score ≥5/10) that were statistically homogenous (*I*^2^ *p* ≥ 0.05). The level of evidence was considered moderate with multiple low-quality studies (two studies with a PEDro score <5/10) that were statistically homogenous and/or one high quality RCT. The level of evidence was considered limited when only one low quality RCT was identified. The level of evidence was conflicting when there were multiple statistically heterogenous studies (*I*^2^ *p* < 0.05).

## Results

### Included Studies

A total of 816 titles were screened in the first search stage, 43 more were included through cross-referencing, and 692 were excluded because they did not concern our research question. The main reasons for exclusion were: absence of WBV treatment (e.g., studies using local vibrations were excluded), measurement of acute effects, no value for dynamic balance, case studies and reviews. Following exclusion, 167 studies were considered for an abstract review. A further 104 were excluded in this second stage because they did not meet the inclusion criteria. Finally, 63 full-text articles were assessed for eligibility with 17 not accepted: five because training lasted <4 weeks, six because they were not RCT, four because there were no walking outcomes, one because it combined WBV training with non-physical therapy and one for comparing WBV training combined with another intervention not provided in the control group (meaning that not only WBV effects were measured). Thus, 46 articles were ultimately included in this systematic review (9, 27–69, 71, 72). A summary of the study selection is provided in Table 1.

**Table 1:** Descriptive checklist of the included studies.

**Article**	**Subjects**	**Interventions**	**Outcomes (only intergroup differences are presented)**
**OLDER ADULTS**			
Lam et al. (50)	73 older adults, 40 women, mean age: 82.3 ± 7.3 years. WBV + exercise (WBV+E): *n* = 25, 12 men, 13 women, mean age 84 years Exercise: *n* = 24, 10 men, 14 women, mean age 82.4 years. Control: *n* = 24, 11 men, 13 women, mean age 80.3 years.	8weeks, 3 times per week. WBV + E*:* Fitvibe medical WBV system (GymnaUniphy NV, Bilzen, Belgium), dynamic exercises, 4 × 1min/1–2min, 30 Hz (weeks 1–4) and 40 Hz (weeks 5–8), 0.9 mm, vertical displacements. Exercise: identical exercise program without WBV. Control: social and recreational activities that only involved the upper limbs.	WBV + E vs. Exercise*:* No significant effect for TUG (SMD = −0.18, 95% CI: −0.74, 0.38) and the 6MWT (SMD = 0.21, 95% CI: −0.35, 0.78).
Wei et al. (69)	80 community dwelling seniors with sarcopenia. Low frequency group: *n* = 20, 7 men, 13 women, mean age 78 years Medium frequency group: *n* = 20, 7 men, 13 women, mean age 75 years High frequency group: *n* = 20, 5 men, 15 women, mean age 74 years Control group: *n* = 20, 5 men, 15 women, mean age 76 years	3 days/week over a 12-week period, WBV: 4 mm for all training groups, knee joint flexed at 60°, Fitvibe excel, GymnaUniphy NV, Bilzen, Belgium, vertical vibrations. Low frequency group: 20Hz × 720s Medium frequency group: 40Hz × 360s High frequency group: 60Hz × 240s Control group: no extra training	Low frequency group vs. control: no significant difference for the TUG test (SMD = −0.22, 95% CI: −0.84, 0.41) Medium frequency group vs. control: no significant difference for the TUG test (SMD = −0.40, 95% CI: −1.03, 0.22) High frequency group vs. control: no significant difference for the TUG test (SMD = −0.30, 95% CI: −0.92, 0.33)
Goudarzian et al. (45)	42 healthy old men. WBV: *n* = 11, mean age 66, 58 years. MT: *n* = 12, mean age 69, 20 years. WBV+MT: *n* = 10, mean age 67, 80 years. Control: *n* = 9, mean age 68, 90 years.	3 times a week, 8 weeks WBV*:* Novotec, Pfor- zheim, Germany, static and dynamic exercises, 6 × 45–85s/45–85s, 30–35 Hz, 5–8 mm, *n* MT: relaxation techniques. WBV+MT: combination of vibration and MT that was the half-time of each protocol. Control: Daily routine.	WBV vs. Control: no difference between group for the TUG test (SMD = −0.60, 95% CI: −1.50, 0.31). Significant improvement of the 10MWT in favor of the WBV (SMD = −1.32, 95% CI: −2.32, −0.33).
Sitjà-Rabert et al. (64)	159 older people, 107 women, 52 men, with a mean age of 82 years. WBV + exercise group: *n* = 81, *n* Exercise group: *n* = 78, *n*	6 weeks, 3 sessions per week. WBV + exercise group*:* Pro5 Airdaptive Model; PowerPlate, Amsterdam, The Netherlands, static/dynamic exercises, *n*, 30–35 Hz, 2–4 mm, *n* Exercise group: same static/dynamic exercises without vibration platform.	WBV + exercise group vs. exercise group: No difference between group for the TUG test (SMD = −0.02, 95% CI: −0.39, 0.34) No difference between group for the Tinetti gait score (SMD = −0.08, 95% CI: −0.44, 0.27)
Santin-Medeiros et al. (62)	37 elderly women, mean age 82.4 years. WBV group: *n* = 25. Control group: *n* = 18.	8-month, 2 sessions per week WBV*:* Fitvibe Excel Pro; GymnaUniphy NV Bilzen, Belgium,18 exercises, 6/session, 1–2 sets/exercise, 30–35/exercise, 6 min-6 min50 s/ session, 20 Hz, 2 mm. Control: maintain their habitual lifestyle	WBV vs. control*:* Groups were statistically different at baseline for the TUG test. WBV: No significant improvement of the TUG test post WBV (SMD = 0.15, 95% CI: −0.49, 0.78).
Buckinx et al. (36)	62 nursing home residents. WBV group: *n* = 31, 11 men, 20 women, mean age 82.2 years. Control group: *n* = 31, 3 men, 27 women, mean age 84.2 years.	6 months, 3 training sessions every week. WBV group*:* Vibrosphère, knees flexed, 5 × 15 s/30 s, 30 Hz, 2 mm, vertical vibrations. Control group: normal daily life.	Lack of data post WBV. WBV vs. control: authors reported no significant inter group difference for the TUG test, Tinetti gait score and for the parameters recorded by the Locometrix (*p* > 0.05).
Lee et al. (53)	55 Elderly Patients with Diabetic Neuropathy WBV + BE group: *n* = 19, 9 men, 10 women, mean age 76.31 years. Balance exercise group (BE): *n* = 18, men:7, women:11, mean age: 74.05 years. Control group: *n* = 18, 8men, 10 women, mean age 75.77 years.	6 weeks, twice per week, same physical therapy. WBV + BE group*:* Galileo 2000, Novotec Medical GmBH, Germany, 3/week, squatting position, 3 × 3min/1-min, 15-30 Hz, 1–3 mm, *n* BE group: strength, balance, and functional mobility training. Control group: *n*	WBV + BE group *vs*. BE group*:* Significant improvement of the TUG test in favor of the WBV group (SMD = −0.72, 95% CI: −1.39, −0.06).
Beaudart et al. (31)	62 nursing home residents. WBV group: *n* = 31, 11 men, 20 women, mean age 82.2 years. Control group: *n* = 31, 4 men, 27 women, mean age 84.2 years.	3 months, 3 training sessions every week. WBV*:* Vibrosphere, static position with a knee flexion, 5 × 15/30 s, 30 Hz, 2mm, vertical vibrations. Control group: requested neither to change their lifestyle during the study nor to get involved in any new type of physical activity.	WBV vs. control: No significant difference between groups for the TUG test (SMD = −0.10, 94% CI: −0.59, 0.40) and the Tinetti test (SMD = 0.30, 95% CI: −0.20, 0.80). Lack of data post WBV for the Locometrix system. The authors reported no significant inter group difference for the parameters recorded by the Locometrix (*p* > 0.05).
Gómez-Cabello et al. (44)	49 non-institutionalized elderly (20 men and 29 women; aged 75.0 ± 4.7 years). WBV: *n* = 24, *n* Control: *n* = 25, *n*	11 weeks, 3 times per week. WBV*:* Pro5 Power plate, London, UK, squat position, 10 × 45/60s, 40Hz, 2mm. Control: not participate in any training. Asked not to change their lifestyle.	WBV vs. control: No difference between group for the 6MWT (SMD = 0.54, 95% CI: −0.03, 1.11).
Bogaerts et al. (32)	111 elderly women over 70 years of age WBV group: *n* = 54 Control group: *n* = 57	6 mouths, 3 sessions per week; WBV group*:* Powerplate, 2–5 dynamic exercises. 4 × 15s/60s (start of the study), 12 × 60s//5s (6mouths). 30-40HZ, 1,6–2,2g, *n* Control group: no training program.	WBV vs. control group*:* No significant difference for the TUG test (SMD = −0.18, 95% CI: −0.55, 0.20) and the 10MWT (SMD = −0.26, 95% CI : −0.63, 0.12) at preferred speed. No significant difference for the TUG test (SMD = −0.31, 95% CI: −0.68, 0.07) and the 10MWT (SMD = −0.10, 95% CI: −0.47, 0.27) at maximum speed.
Mikhael et al. (56)	19 older adults mean age 64, 4 years (range 50–80). WBV with flexed knees (FK): *n* = 6, 4 men, 2 women, mean age 63.3 years WBV with locked knees (LK): *n* = 5, 3 men, 2 women, mean age 69 years Sham: *n* = 8, 4 men, 4 women, mean age 62.3 years	20 min, 3 days per week, 3 months, static exercises, 39 × 1min/1min, 12 Hz, 1 mm WBV with FK: vibration platform engineered by Australian Catholic University (2004), knee angle at 20 WBV with LK: lock knees Sham*:* The amplitude was set to 0 mm, giving 0 g magnitude.	Lack of data post WBV. The authors reported no between groups difference after WBV for the 6MWT (*p* = 0.61), habitual and maximal gait velocities (*p* = 0.80 and *p* = 0.58, respectively).
Machado et al. (55)	26 community-dwelling elderly women WBV: *n* = 13, mean age 79.3 years Control: n = 13, mean age 76.2 years	3–5 times a week, 10 weeks WBV: Fitvibe, GymnaUniphy NV, Bilzen, Belgium, half squat, deep squat, wide stance squat, calves, 1–2 sets/exercise, 30–60 s/120–180 s, 2–4 mm, 20–40 Hz, increased progressively, *n* Control: requested to do not change their lifestyle during the study	Lack of data post WBV. The authors reported a significant improvement of the TUG test post WBV(*p* < 0.05) but no significant difference between groups (*p* > 0.05).
Furness and Maschette. (40)	73 older adults, 38 females and 35 males, mean age 72 ± 8 years 1 WBV session per week: *n* 2 WBV sessions per week: *n* 3 WBV sessions per week: *n* Control group: *n*	0, 1, 2, or 3 times a week, 6 weeks. WBV interventions: *n*, static, knees flexes at 110°, 5 × 1min/1min, 15–25 Hz, 0,5mm, vertical vibrations. Control group*:* The zero group did not participate in any WBV sessions.	1 WBV vs. control: No significant between group difference for the TUG test (SMD = 0.57, 95% CI : −0.10, 1.24) 2 WBV vs. control: No significant between group difference for the TUG test (SMD = 0.57, 95% CI: −0.10, 1.24) 3 WBV vs. control: No significant between group difference for the TUG test (SMD = −0.45, 95% CI: −1.11, 0.20)
Rees et al. (59)	43 older adults, untrained, 23 men and 20 women WBV group: *n* = 15, mean age 74.5 years Exercise group: *n* = 13, mean age 73.1 years Control group: *n* = 15, mean age 73.1 year	3 sessions a week, 8weeks, low- intensity walking at least 3 times a week WBV group*:* Novotec, Pforzheim, Germany, static and dynamic exercises, 6 × 45–80s/45–80s, 26 Hz, 5–8 mm, increased progressively, vertical displacements. Exercise group: same exercises without WBV. Control group: low intensity walking	WBV vs. exercise group: No significant difference between groups for the TUG test (SMD = −0.35, 95% CI: −1.10, 0.40) and the 10MWT (SMD = −0.25 95% CI: −0.99, 0.50). WBV vs. control: no significant difference between group for the TUG test (SMD = −0.22, 95% CI: −0.75, 0.31) and the 10MWT test (SMD = −0.29, 95% CI: −1.01, 0.43).
Bautmans et al. (30)	24 older adults, nursing home residents. WBV: *n* = 13, 5 men, 8 women, mean age 76.6 years. Sham: *n* = 11, 4 men, 7 women, mean age 78.6 years.	3 times weekly, 6 weeks WBV: Power- Plate, Badhoevedorp, The Netherlands, 2–4 static lower limb exercises/sessions, 1–3 × 30–60s/30–60s, 35–40 Hz, 2–5 mm, increased progressively, vertical vibrations. Sham: same exercise program on the vibration plate, but without vertical vibrations.	WBV vs. sham*:* No significant difference between groups for the TUG test (SMD = −0.38, 95% CI: −1.25, 0.48) and the Tinetti test (SMD = 0.00, 95% CI:−0.86, 0.86).
Bruyere et al. (9)	42 older adults, nursing home residents WBV + Physical therapy: *n* = 22, 4 men, 18 women, mean age 84.5 years Physical therapy: *n* = 20, 7 men, 13 women, mean age 78.9 years	3 times a week, 6 weeks, same PT 10 min WBV + Physical therapy: *n* static exercise, 4 × 60 s/90 s, 10–26 Hz, 3–7 mm, vertical vibrations. Physical therapy*:* PT only.	Lack of data post WBV. Groups were statistically different at baseline for the TUG test (*p* = 0.04). The authors reported a significant decrease of 11.0 ± 8.6 s post WBV for the TUG test and an increase of 3.5 ± 2.1 points post WBV for the Tinetti gait score.
**PATIENTS WITH CHRONIC OBSTRUCTIVE PULMONARY DISEASE (COPD)**			
Spielmanns et al. (71)	28 subjects with COPD stage II-IV. WBVT group: *n* = 12, 8 men, 4 women, mean age 62.4 years. Conventional training group (CTG): *n* = 16, 9 men, 7 women, mean age 68 years.	3 months, 2 sessions/week, same resistance, and endurance training. WBVT group*:* Galileo vibration platform (No- votec Medical, Pforzheim, Germany), 3 × 20 squat repetitions, 24–26 Hz,3 mm, side-alternating vibration. CTG: same of squat exercises but without WBVT.	WBV vs. control: no significant difference between groups for the 6MWT (SMD = 0.72, 95% CI:−0.05, 1.50).
Spielmanns et al. (65)	29 subjects with stable COPD in stage I to III WB group: *n* = 14, 7 men, 7 women, mean age 69 years. Calisthenics group: *n* = 14, 7 men, 7 women mean age 70 years.	3 months, twice per week. WBV group*:* Galileo, Novotec Medical, Pforzheim, Germany, isometric squat position, 3 × 2 min/2 min, 6 −10 Hz, 4–6mm, side-alternating vibration. Calisthenics group: relaxation, breathing retraining, calisthenics exercises.	WBV vs. Calistenic: no significant difference between groups for the 6MWT (SMD = 0.54, 95% CI:−0.23, 1.32).
Salhi et al. (61)	62 patients with COPD WBV-group: *n* = 31, 21 men, 10 women, mean age 58 years. Conventional resistance training (RT): *n* = 31, 23 men, 8 women, mean age 63 years.	12 weeks, 3 times a week, same pulmonary rehabilitation program. WBV-group*:* FITVIBE, Gymna, Belgium, 8 upper and lower body exercises, 1–3 sets/exercise, 30–60s/*n* 27 Hz, 2 mm, vertical vibrations. RT: lower and upper body exercises, 3 × 10repetitions	WBV vs. RT: no significant difference between groups for the 6MWT (SMD = −0.24, 95% CI: −0.79, 0.31)
Pleguezuelos et al. (57)	51 stable male patients with COPD Whole Body Vibration Training Group: *n* = 26, mean age 68.4 years. Control group: *n* = 25, mean age 71.3 years.	6 weeks, 3 sessions per week, regular prescribed medical treatment. WBVTG*:* Gymnauni phy. Nv. Pasweg 6a 3740 Bilzen, Belgium, squatting position, 6 × 30s/60s, 35Hz and 2mm, vertical vibrations. Control Group: general recommendations about physical activity and lifestyle.	WBV vs. control: no significant difference between groups for the 6MWT (SMD = 2.59, 95% CI 1.83, 3.35).
**STROKE**			
Alp et al. (28)	21 post stroke patients WBV: (*n* = 10), 10 men, 0 women, mean age 61.20 ± 11.043 years. Control group: (*n* = 11) 9 men, 2 women, mean age 62.9 ± 8 years	4 weeks, 3 days a week, stretching and active range of motion exercises on the hemiplegic lower extremity for 15 min. WBV*:* Compex Winplate by Uniphy Elektromedizin GmbH and CoKG, tiptoes, 3 × 10s/3–20 s, 5min, 40 Hz, 4 mm,*n* Control group: same exercises, no vibration.	Lack of data post WBV. The groups were statistically different at baseline for the 10MWT (*p* < 0.001). The authors reported a significant improvement of the 10MWT in favor of the WBV group (*p* < 0.001).
Choi et al. (72)	30 individuals who presented with a gait deviation after a first stroke (>6 months). WBV-Treadmill Training (TT): *n* = 15, 8 men, 7 women, mean age 51.93 years. TT group: *n* = 15, 11 men, 4 women, mean age 53.67 years.	6 weeks, 3 times a week, 20 min of TT for both groups. WBV- TT*:* Galileo 2000, (Novotec, Germany, 2011), dynamic exercises, 6 × 45s/1min20-30 HZ, 3 mm, side- alternating vibration. TT group: same exercises on the platform without vibration.	No significant difference between group for the Walking speed (SMD = 0.32, 95% CI: −0.40, 1.04) and stride length (SMD = 0.50, 95% CI: −0.23, 1.23).
Choi et al. (38)	22 individuals who were diagnosed with strokes at least 6 months prior to the study. WBV group: *n* = 11, 8 males, 3 females, mean age 50.9 years. Control group: *n* = 11, 7 males, 4 females, mean age 52.2 years.	4 weeks, 5 times per week. WBV group*:* Galileo tilt table (Novotec Medical, Germany), squat posture, 10 min/session, 25 Hz, 5 mm,*n* Control group: 30 min of Neuro-developmental treatment as the experimental group.	WBV vs. control group: no significant difference between group for the TUG test (SMD = −0.50, 95% CI : −1.35, 0.35).
Liao et al. (54)	84 individuals with hemispheric stroke persisting for more than 6 months before the time of enrolment low-intensity WBV group (LWBV): *n* = 28, 20 men, 8 women, mean age 60.9 years High-intensity WBV group (HWBV): *n* = 28, 18 men, 10 women, mean age 62.9 years Control (CON): *n* = 28, 24 men, 4 women, mean age 59.8 years.	3 times a week, 30 sessions, same dynamic and static exercises, Gymna Fitvibe Medical System, Gymna Uniphy Pasweg, Bilzen, Belgium, synchronous vibrations. LWBV: 20 Hz, 1 mm HWBV: 30 Hz, 1 mm Control (CON): standing on the same WBV platform turned off.	LWBV vs. control: no significant difference between group for the 6MWT (SMD = 0.05 95% CI:−0.47, 0.58) and for TUG test (SMD = −0.10 95% CI: −0.62, 0.43). HWBV vs. Control: no significant difference between group for the 6MWT (SMD = −0.03, 95% CI−0.55, 0.50) and for TUG test (SMD = −0.25, 95% CI: −0.77, 0.28).
Lau et al. (51)	82 chronic stroke patients. WBV group: *n* = 41, 26 men, 15 women, mean age 57.3 years. Control group: *n* = 41, 32 men, 9 women, mean age 57.4 years.	8 weeks, 3 times a week. WBV*:* Jet-Vibe System (Danil SMC Co. Ltd., Seoul, South Korea), dynamic leg exercises, 6 exercises, 9–15 × 1,5-2, 5min/*n*, 20–30 Hz, 0.44–0.60 mm, vertical vibrations. Control group: same exercises without vibration.	WBV vs. control: no significant improvement of the 6MWT (SMD = −0.22 95% CI = −0.66, 0.21) and the 10MWT (SMD = 0.39 95% CI: −0.05, 0.83).
Brogårdh et al. (35)	31 individuals with chronic stroke. WBV: *n* = 16, 13 men, 3 women, mean age 61.3 years. Control group: *n* = 15, 12 men, 3 women, mean age 63.9 years.	6 weeks, 2 sessions/week. WBV training*:* Xrsize, static position knee flexed,4–12 × 40–60s/1min, 25Hz, 3.75 mm, vertical vibrations. Control group: placebo vibrating platform (25Hz,0.2 mm amplitude).	WBV vs. control group: groups were different at baseline for the TUG test and the 6MWT. The authors reported significant improvements in both outcomes after WBV (*p* < 0.05)
van Nes Ilse et al. (67)	53 post-stoke patients WBV group: *n* = 27, 16 males, 11 females, mean age of 59.7 years. Exercise therapy on music group: *n* = 26, 14 males, 12 females, mean age of 62.6 years.	6 weeks, 5 days per week, physical therapy WBV group*:* Galileo 900, Galileo 2000, Enschede, The Netherlands, squat position hips and knees slightly flexed, 4 × 45 s/60 s, 30 Hz, 3 mm, Side-alternating vibration. Exercise therapy on music group: same standing position, exercises and relaxation.	WBV vs. exercise therapy on music group: no significant difference between groups (SMD = 0.00, 95% CI: −0.54, 0.54).
**KNEE OSTEOARTHRITIS**			
Bokaeian et al. (33)	28 patients with knee osteoarthritis. WBV + Strength Training (ST): *n* = 15, 0 men, mean age 51.8 years Strength Training: *n* = 13, 2 men, 11 women, mean age 54 years.	8 weeks, 3 times a week, both groups received same PT and strengthening exercises protocol. WBV + ST group*:* Fitvibe device (Italy), bent knees, 6–9 × 30–70 s/30–70 s, 25–30 HZ, 2 mm, progressively increased, vertical vibrations. Strength Training: flexion and extension exercise of knee joint, 3 sets, 10 RM, progressive load.	Lack of data post WBV. The authors reported significant improvements for the 2MWT, 50FWT and TUGT in favor of the WBV + ST group (*p* = 0.009).
Wang et al. (68)	49 patients with knee osteoarthritis. Whole Body Vibration Exercise + Quadriceps Resistance Exercise group: *n* = 49, 13 men, 36 women, mean age 61.2 years. Quadriceps Resistance Exercise only group: *n* = 50, 15 men, 35, women, mean age 61.5 years.	24 weeks, 5 days/week. WBV + QRE*:* My7TM model Personal Plate, Power Plate, USA, knees slightly flexed, 30 × 60 s/60 s, 35 Hz, 4–6 mm,*n* Quadriceps Resistance Exercise: static and dynamic exercises.	WBV + QRE vs. QRT: significant improvement in favor of the WBV+QRE group for the TUG test (SMD = −3.11 95% CI: −3.71, −2.52) and the 6MWT (SMD = 1.68, 95% CI : 1.22, 2.14)
Simão et al. (63)	31 elderly subjects with knee osteoarthritis. WBV group: *n* = 10, mean age 75 years. Squat group: *n* = 10, mean age 73.4 years. Control group: *n* = 11, mean age 71 years.	12 weeks, 3 times per week. WBV group*:* FitVibe, squat exercise, 6–8 × 20–40 s/20–40 s, 35–40 HZ, 4 mm, vertical vibrations. Squat group: 3s of isometric contraction. Control group: without intervention. No change their lifestyle.	WBV vs. Control group: no significant difference between groups for the 6MWT (SMD = 0.56, 95% CI: −0.26, 1.37) and gait speed (SMD = 0.39, 95% CI−0.42, 1.20).
Avelar et al. (29)	21 elderly patients with knee osteoarthritis. WBV group: *n* = 11, mean age 75 years. Control group: *n* = 10, mean age 71 years.	12 weeks, 3 times per week. WBV*:* FitVibe, GymnaUniphy NV, Bilzen, Belgium, squat training with WBV, 6–8 × 20–40 s/20–40 s, 35 Hz−40 Hz, 4 mm, *n* Control: squat training without vibration.	WBV vs. control group: no significant difference between groups for the TUG test (SMD = 0.06, 95% CI: −0.80, 0.91). Significant improvement of the 6MWT in favor of the WBV group (SMD = 1.49, 95% CI: 0.49, 2.48).
**POSTMENOPAUSAL WOMEN**			
Sucuoglu et al. (66)	42 postmenopausal women patients WBV- Balance Coordination Exercise (BCE) group: *n* = 21, mean age 56 years. BCE group: *n* = 26, mean age 58.76 years.	4 weeks, 10 sessions per week, identical BCE programs. WBV-BCE group*:* Power Plate (Performance Health Systems UK Ltd, London, UK), 3 static positions, 2 × 30 s/60 s 30–35 Hz. 5 sessions per week, vertical vibrations. BCE group: 20-min exercise sessions at home, twice per day.	Significant difference at baseline between groups for the TUG test. The authors reported significant improvement compared with pretreatment values in both groups (*p* < 0.005).
Iwamoto et al. (48)	52 ambulatory postmenopausal women with osteoporosis. WBV group: *n* = 26, mean age 72.4 years. Control group: *n* = 26, mean age 76 years.	6 months, *n* WBV group*:* Galileo machine (G- 900; Novotec, Pforzheim, Germany), bent knees, 4 min, 20 HZ, *n*, side to side alternating vibrations. Control group: no exercise.	Lack of data post WBV. The authors reported no significant difference between groups for the TUG test (*p* > 0.05) but significant improvement in favor of the WBV group for the 10MWT (*p* < 0.05).
Raimundo et al. (58)	27 postmenopausal women WBV: *n* = 14, mean age 66 years Walk based program: *n* = 13, mean age 66 years.	3 times a week, 8 months WBV*:* Galileo 154 2000, Novotec GmbH, Pforzheim, Germany, static knees flexed at 120°, 3–6 × 1 min/1 min, 12,6 Hz, 6 mm, increased each week, side-alternating oscillations. Walk based program: 2 × 25 min of walk, 70–75% HRmax	Lack of data post WBV. The authors reported a significant improvement of the 10MWT post WBV (*p* = 0.006).
**MULTIPLE SCLEROSIS**			
Ebrahimi et al. (39)	34 multiple sclerosis patients with mild to moderate disability WBV group: *n* = 17, 5 men, 12 women, mean age 37.06 years. Control group: *n* = 17 4 men, 13 women, mean age 40.75 years.	10 weeks, 3 times a week. WBV group: *n*, static positions, 15 × 30s−2 min/30 s−5 min, 2–20 Hz, 2 mm, *n* Control group: continued their normal life	WBV vs. control group: no significant difference between groups for the TUG test (SMD = −0.47, 95% CI: −1.20, 0.26). Significant improvement in favor of the WBV group for the 10MWT (SMD = −1.05, 95% CI: −1.82, −0.28) and the 6MWT (SMD = 1.22, 95% CI: 0.43, 2.01).
Broekmans et al. (34)	25 ambulatory community- based patients with multiple sclerosis. WBV: *n* = 11, 7 men, 4 women, mean age 46.1 years Control: *n* = 14, 11 men, 3 women, mean age 49.7 years	5 sessions per 2-week cycle, 20 weeks WBV*:* Alpha Vibe^®^ Nijverdal, The Netherlands, 2–5 static and dynamic leg squats and lunges, 1–3 × 30–60 s/30–120 s, 20–45 Hz, 2.5 mm, increased progressively, vertical vibrations. Control: maintain their usual lifestyle	Groups were statistically different at baseline for the TUG test and 2MWT. The authors reported no significant effects in both groups for the TUG test (*p* = 0.26) and the 2MWT (*p* = 0.25).
**OTHER PATHOLOGIES IN ADULTS**			
In et al. (47)	28 patients who were diagnosed with incomplete cervical spinal injury WBV group: *n* = 14, 9 men, 5 women, mean age 46.1 ± 9.8 years Control group: *n* = 14, 10 men, 4 women, mean age 49.9 ± 9.3 years	8 weeks, 5 days a week, twice a day, conventional physical therapy WBV group: TT2590X7, TurboSonic Co., South Korea, semi-squat with slight flexion (140°) at hips, knees and ankles, 4 × 45 s/1 min, 30Hz, 2–4 mm, vertical vibrations. Control group: 16 min of placebo WBV and 30 min of physical therapy.	WBV vs. Control group*:* No significant difference between groups for the TUG test (SMD = −0.64, 95 CI: −1.40, 0.13) and the 10MWT (SMD = −0.23, 95% CI:-0.97, 0.52).
Gerhardt et al. (42)	22 adult patients with stable, symptomatic pulmonary arterial hypertension (PAH). WBV group: *n* = 11, 7 men, 4 women, mean age 65.1 years. Control group: *n* = 11, 6 men, 5 women, 46 years.	4 weeks, 16 sessions. WBV group: Galileo MedM plat- form (Novotec Medical GmbH, Pforzheim, Germany), specific coordination exercises, *n*, 20 Hz, 20 mm, side alternating vibrations. Control group: received WBV in a second phase.	Lack of data post WBV. The authors indicated that WBV was associated with a significant improvement of the 6MWD versus baseline of +38.6 ±6.6 m (*p* < 0.001)
Gloeckl et al. (43)	83 patients after lung transplantation. WBVT group: *n* = 34, 16 men, 18 women, mean age 56 years. Control group: *n* = 36, 22 men, 14 women, mean age 56 years.	4 weeks, 3 times per week, same pulmonary rehabilitation program. WBVT group*:* GALILEO, Novotec Medical GmbH, Pforzheim, Germany, squat exercises, 4 × 2min/4min, 24–26 Hz, 6 mm, side alternating vibrations. Control group: same squat training on the floor.	lack of data post WBV The authors reported a between group difference of 28 m (95%CI: 3 m to 54 m, *p* = 0.029) significantly different in favor of WBVT.
Gaßner et al. (41)	17 participants diagnosed with idiopathic Parkinson's disease. WBV group: *n* = 8, 6 men, 2 women, mean age 71.4 years. Placebo group: *n* = 9, 7 men, 2 women, mean age 68.2 years.	5 weeks, 2–3 times a week. WBV group*:* SRT Zeptor Medical plus noise, static position, 5 × 60 s/60 s, knees slightly bents, 6 Hz, 3 mm, *n* Placebo group: stood on the vibration platform in the same basic position.	WBV vs. placebo group: no significant difference between group for the TUG test (SMD = −0.37, 95% CI: −1.34, 0.59), velocity (SMD = −0.21, 95% CI: −1.17, 0.74) and step length (SMD = 0.14, 95% CI: −0.81, 1.09).
Johnson et al. (49)	16 individuals, 3–6 weeks post total knee arthroplasty WBV: *n* = 8, 6 men, 2 women, mean age 67 years Traditional Progressive Resistance Exercise (TPRT): *n* = 8, 4 men, 4 women, mean age 68.5 years	3 session a week, 4 weeks WBV: Power Plate, Badhoevendorp, The Nertherlands, static and dynamic exercises, 4–6 exercises/session, 1–3 set/exercise, 30–60 s/*n*,35 Hz, 2–5 mm, *n* TPRE: 1–3 SET of 10 REP for strengthening exercises for lower limbs, exercises were progressed once the patient could complete the exercise	WBV vs. TRPE: no significant difference for the TUG test (SMD = −0.59, 95% CI: −1.59, 0.42).
Ahlborg et al. (27)	14 persons with cerebral palsy, spastic diplegia WBV: *n* = 7, 4 men, 3 women, mean age 32 years Resistance training: *n* = 7, 4 men, 3 women, mean age 30 years	Three times weekly, 8 weeks, same warming up and stretching WBV: NEMES-LSC (Nemesis BV, Hengelo, The Netherlands), standing position, hips and knees in 50° of flexion, 1–4 × 30–110 s/15–120 s, increased progressively, 11 levels of intensity, 25–40 Hz, 7/10 on the Borg Scale, *n* Resistance training: leg press, 3 SET of 10–15 REP, progressive load.	WBV vs. RT: no significant difference between groups for the TUG test (SMD = 0.28, 95% CI: −0.77, 1.34).
**OTHER PATHOLOGIES IN CHILDREN**			
Högler et al. (46)	24 children (5–16 years) with clinically mild to moderate osteogenesis imperfecta. WBV training: *n* = 12, 6 men, 6 women, mean age 9.38 years. Control group: *n* = 12, 6 men, 6 women, mean age 6.49 years.	5 months, twice-daily, home use WBV training: Galileo MTM, Novotec Medical, Pforzheim, Germany), static and dynamic exercises, 3 × 3 min/3 min, 20–25 Hz, side alternating vibrations. Control group: continued to receive regular care.	Lack of data post WBV. The authors reported no significant difference between groups for the 6MWT (*p* = 0.278)
Cheng et al. (37)	16 children with cerebral palsy, 8 boys and 8 girls, mean age 9.2 years. WBV group: *n* = 8, *n* Control group: *n* = 8, *n*	8-week WMV intervention followed by an 8-week control condition, with a 4-week rest (crossover study). WBV*:* AV-001A, Body Green, Taipei, Taiwan, static position, 10 min, 20 Hz, 2 mm, vertical vibrations. Control: same procedure with the machine turned off.	Lack of data post WBV. The authors reported a significant difference between the treatment and control condition for the 6MWT (*p* = 0.005).
Lee and Chon. (52)	30 patients with either the spastic diplegia or quadriplegia forms of cerebral palsy WBV group: *n* = 15, 6 men, 9 women, mean age 10 years. Control group: *n* = 15, 9 men, 6 women, mean age 9.66.	8 weeks, 3 days per week, conventional PT. WBV*:* Galileo system (Novotec Medical GmbH, Pforzheim, Germany), squat position, 6 × 3 min/3 min, 5–25 Hz, 1–9 mm, side alternating vibrations. Control group: conventional physical therapy training.	WBV vs. control group: Significant improvement in favor of the WBV group for the gait speed (SMD = 1.41, 95% CI: 0.60, 2.22) and stride length (SMD = 0.91, 95% CI: 0.15, 1.67)
Ruck et al. (60)	20 children with cerebral palsy WBV: *n* = 10, 8 boys, 2 girls mean age 8.3 years Control: *n* = 10, 6 boys, 4 girls, mean age 8.1 years	Physiotherapy according to the established school program, 6 months, 5 days per week WBV: Vibraflex Home Edition II^®^, Orthometrix Inc, White Plains, NY. Outside of North America, Galileo Basic, knees and hips flexed 10–45°, dynamic exercises, 3 × 3 min/3 min, 12–18 Hz, 2–6 mm, side to side alternating vertical vibrations. Control: Physiotherapy only	Lack of data post WBV. The authors reported a significant improvement of the 10MWT in favor of the WBV (*p* = 0.03).

*WBV, Whole body vibration; TUG, Timed up and go test; 6MWT, 6-minute walk test; 2MWT, 2-minute walk test; 50FWT, 50-feet walk test; 10MWT, 10-meter walk test*.

### Characteristics of the Populations

A total of 2 029 patients took part in the 46 studies selected in this review (see Table 1). The sample size ranged from 14 to 159 participants, with a mean age of 60.9 ± 20.0 years, varying from 7.9 years to 83.2 years. With regard to the adult population, 16 studies evaluated the effects of WBV in the elderly (*n* = 59.8 ± 35.4 subjects) (9, 30–32, 36, 40, 44, 45, 50, 53, 55, 56, 59, 62, 64, 69), four in patients with Chronic Obstructive Pulmonary Disease (COPD) (*n* = 42.5 ± 16.7 subjects) (57, 61, 65, 71), seven in patients with stroke (*n* = 46.1 ± 27.2 subjects) (28, 35, 38, 51, 54, 67, 72), four in patients with osteoarthritis (OA) (*n* = 32.2 ± 11.9 subjects) (29, 33, 63, 68), three in postmenopausal women (*n* = 40.3 ± 12.5 subjects) (48, 58, 66), two in patients with multiple sclerosis (*n* = 29.5 ± 6.3 subjects) (34, 39) and one in patients with the following pathologies: incomplete cervical spinal injury (47), pulmonary arterial hypertension (42), lung transplantation (43), idiopathic Parkinson's disease (41), total knee arthroplasty (49) and cerebral palsy (27) (*n* = 30.0 ± 26.4 subjects). With regard to the child population, two studies evaluated the effects of WBV in cerebral palsy (37, 60), one in patients with osteogenesis imperfect (46) and one in patients with spastic diplegia or quadriplegia forms of cerebral palsy (52) (*n* = 22.5 ± 5.9 subjects). Most of the studies included both males and females, except for nine studies that either did not mention the participants' gender or selected only males or females (including the three studies on post-menopausal women). Most of the studies clearly explained their eligibility criteria and had similar baselines (no significant differences between groups in any outcomes before the intervention) in their groups, except in 10 articles.

### Training Protocols

The duration of the WBV training interventions ranged from four to 32 weeks, with between two and five sessions per week, with a mean of 3.1 ± 0.8 (three sessions per week in 31 of the 46 selected articles). The frequency and amplitude used in the training sessions ranged from 2 to 45 Hz and from 0.44 to 20 mm, respectively. The intensity of the training sessions, by frequency and/or amplitude, was progressively increased in 30 studies, and remained unchanged in the other selected studies. Some WBV platforms delivered the vibrations alternating between the right and the left foot, while the right and left foot moved up and down at the same time in other vibration plates (70). Synchronous vibrations were delivered in 20 studies, side-alternating vibrations were used in 11 studies, while 15 studies did not mention the type of vibration in their intervention method.

For the groups that were exposed to WBV training (interventions groups), vibrations were delivered while participants stood in static positions (e.g., squat or lunge positions) in 27 studies and dynamic exercises were provided in 11 studies. In the remaining eight studies, both static and dynamic exercises were combined during the WBV training sessions. The number of WBV sets per training session ranged between 1 and 135. The duration of the vibration sets ranged from 10 s to 3 min, with a between-sets resting time ranging between 3 s and 5 min. For the groups not exposed to WBV training interventions (control groups), participants performed strengthening and balance exercises without WBV in fourteen studies, had no intervention and were asked to maintain their habitual lifestyle in sixteen studies, were exposed to a sham intervention in six studies, continued to follow their conventional physiotherapy in four studies, received relaxation exercises in four studies and performed walking training sessions in two studies.

### Gait Motor Outcomes

The “Timed Up-and-Go” (TUG) test and the “six-minute walking test” (6MWT) were the clinical outcomes most frequently used to assess gait (in 29 and 18 studies, respectively). The “ten-meter walking test” (10MWT) was used in 10 studies to assess gait velocity. Walking speed was also evaluated using biomechanical and kinematic assessments (e.g., walking on a platform or camera motion analysis) in six studies. Other temporal and spatial parameters such as time of swing phase and stance phase, stride length and step length were presented in only two studies. Gait quality was assessed using the gait score of the Tinetti test in five studies. Finally, other outcomes were used once in all 46 studies: the “functional ambulation categories test” with stroke patients, the “50-foot walking test” with knee OA patients, the “25-foot walking test” with multiple sclerosis patients, the “two-minute walking test” with knee OA patients, and the time to walk four meters in postmenopausal women. A summary of the primary outcomes related to gait is provided in Table 1.

### Quality Assessment

The results from the quality assessments for each of the studies for respective quality indexes are provided in Table 2. According to the PEDro Scale, 40 studies obtained a high-quality methodology score while six studies were rated as low quality.

**Table 2:** Quality assessment with the PEDro scale.

**Article**	**Items by number on the PEDro scale**											**Total score**	**Subjects**
	**1**	**2**	**3**	**4**	**5**	**6**	**7**	**8**	**9**	**10**	**11**		
Lam et al. (50)	y	y	y	y	n	n	y	n	n	y	y	6	Older adults
Wei et al. (69)	y	y	n	y	n	n	n	y	y	y	y	6	
Goudarzian et al. (45)	y	y	n	y	n	n	n	y	n	y	y	5	
Sitjà-Rabert et al. (64)	y	y	y	n	n	n	y	n	y	y	y	6	
Santin-Medeiros et al. (62)	y	y	n	n	n	n	n	n	y	y	y	4	
Buckinx et al. (36)	y	y	n	n	n	n	y	n	y	y	y	5	
Lee et al. (53)	y	y	n	y	n	n	n	y	n	y	y	5	
Beaudart et al. (31)	y	y	n	y	n	n	y	y	y	y	y	7	
Gómez-Cabello et al. (44)	n	y	n	n	n	n	n	y	y	y	y	5	
Bogaerts, et al. (32)	y	y	y	y	n	n	n	y	n	y	y	6	
Machado et al. (55)	y	y	n	n	n	n	y	y	n	y	y	5	
Mikhael et al. (56)	y	y	y	y	y	n	y	n	n	y	y	7	
Furness and Maschette (40)	y	y	n	n	n	n	n	y	y	y	y	5	
Rees et al. (59)	y	y	n	y	n	n	n	y	n	y	y	5	
Bautmans et al. (30)	y	y	y	y	y	n	y	y	n	y	y	8	
Bruyere et al. (9)	y	y	n	n	n	n	n	y	y	y	y	5	
Spielmanns et al. (71)	y	y	y	y	n	n	n	n	n	y	y	5	COPD
Spielmanns et al. (65)	y	y	n	y	n	n	n	y	n	y	y	5	
Salhi et al. (61)	y	y	y	y	n	n	n	n	n	y	y	6	
Pleguezuelos et al. (57)	y	y	n	y	n	n	y	n	n	y	y	5	
Alp et al. (28)	y	y	y	n	n	n	y	y	n	y	y	6	Stroke
Choi et al. (72)	y	y	y	y	n	n	y	y	y	y	y	8	
Choi et al. (38)	y	y	n	y	n	n	n	n	n	y	y	4	
Liao et al. (54)	y	y	y	y	n	n	y	y	y	y	y	8	
Lau et al. (51)	y	y	y	y	n	n	y	y	y	y	y	8	
Brogårdh et al. (35)	y	y	y	y	y	n	y	y	y	y	y	9	
van Nes Ilse et al. (67)	y	y	y	n	y	n	y	y	y	y	y	8	
Bokaeian et al. (33)	y	y	y	y	n	n	n	y	n	y	y	6	Knee OA
Wang et al. (68)	y	y	y	y	n	n	y	y	y	y	y	8	
Simão et al. (63)	y	y	y	y	n	n	y	y	n	y	y	7	
Avelar et al. (29)	y	y	n	y	n	n	n	y	n	y	y	5	
Sucuoglu al. (66)	y	y	n	y	n	n	n	n	n	y	y	4	Postmenauposal women
Iwamoto et al. (48)	y	y	n	y	n	n	n	y	y	y	y	6	
Raimundo et al. (58)	y	y	n	y	n	n	n	n	n	y	y	4	
Ebrahimi et al. (39)	y	y	n	y	n	n	n	y	n	y	y	5	Multiple sclerosis
Broekmans et al. (34)	y	y	y	y	n	n	y	y	n	y	y	7	
In et al. (47)	y	y	y	y	y	n	y	y	n	y	y	8	Other pathologies in adults
Gerhardt et al. (42)	y	y	n	y	n	n	n	n	n	y	y	4	
Gloeckl et al. (43)	y	y	y	y	n	n	y	n	n	y	y	6	
Gaßner et al. (41)	y	y	n	y	n	n	n	y	n	y	y	5	
Johnson et al. (49)	y	y	n	n	n	n	n	n	n	y	y	3	
Ahlborg et al. (27)	y	y	n	y	n	n	n	y	y	y	y	6	
Högler et al. (46)	y	y	y	y	n	n	n	n	n	y	y	5	Other pathologies in children
Cheng et al. (37)	y	y	n	y	n	n	n	y	y	y	y	6	
Lee et al. (53)	y	y	y	y	n	n	y	y	y	y	y	8	
Ruck et al. (60)	y	y	y	y	n	n	n	n	n	y	y	5	

*n, criterion not fulfilled; y, criterion fulfilled; 1, eligibility criteria were specified; 2, subjects were randomly allocated to groups or to a treatment order; 3, allocation was concealed; 4, the groups were similar at baseline; 5, there was blinding of all subjects; 6, there was blinding of all therapists; 7, there was blinding of all assessors; 8, measures of at least one key outcome were obtained from more than 85% of the subjects who were initially allocated to groups; 9, intention-to-treat analysis was performed on all subjects who received the treatment or control condition as allocated; 10, the results of between-group statistical comparisons are reported for at least one key outcome; 11, the study provides both point measures and measures of variability for at least one key outcome; total score, each satisfied item (except the first) contributes 1 point to the total score, yielding a PEDro scale score that can range from 0 to 10. B, the level of evidence was B (randomized control trials that lacked double-blinding)*.

The mean score was 5.8 ± 1.4 with a median of 5.5 and a range of scores from 3 to 9. The highest-quality methodology scores were found in the articles concerning stroke patients, with a mean score of 7.2 ± 1.7. The poorest methodological quality was found for postmenopausal women with a mean score of 4.7 ± 1.1.

### Studies Included for Meta-Analysis

A total of 25 studies were included in statistical analysis. Eleven studies were included for meta-analysis in the elderly (30–32, 40, 44, 45, 50, 53, 59, 64, 69), four studies for COPD patients(57, 61, 65, 71), four studies for stroke patients (35, 38, 51, 54), four studies for patients with knee OA (29, 33, 63, 68) and two studies for patients with Multiple Sclerosis (MS) (3, 34).

### Results Ranked According to Aging and Pathology

#### Elderly Subjects

Sixteen studies examined the effect of WBV on elderly subjects(9, 30–32, 36, 40, 44, 45, 50, 53, 55, 56, 59, 62, 64, 69). The studies had an average PEDro score of 5.5 ± 1.0. The sample size ranged from 19 to 159 participants with a mean age of 76.5 ± 5.8 years. Most of the studies included both men and women except for three with women only (32, 55, 62) and one with only men (45). Only one study failed to mention the eligibility criteria (44) and seven studies exhibited heterogeneity in their baselines (9, 36, 40, 44, 55, 62, 64). Training duration varied from 6 weeks to 8 months. Fifteen studies had a frequency of three sessions per week (9, 30–32, 36, 40, 44, 45, 50, 53, 55, 56, 59, 64, 69) while one study involved two sessions per week (62). The frequency and amplitude of platform vibrations varied from 10 to 40 Hz and 0.5 to 8 mm, respectively. Intensity was progressively increased in 11 studies (9, 30, 32, 40, 45, 50, 53, 55, 59, 62, 64). Eight studies used synchronous vibrations (9, 30, 31, 36, 40, 50, 59, 69) while the other eight studies (32, 44, 45, 53, 55, 56, 62, 64) did not mention the type of vibrations delivered by their devices. The number of vibration bouts delivered per session varied from two to 39 sets with a period lasting between 15 sand 3 min each. Resting time was between 5 s and 5 min. In nine protocols (9, 30, 31, 36, 40, 44, 53, 56, 69), the subjects maintained a static position, while they performed dynamic exercises in three studies (32, 50, 55), or both in four studies (45, 59, 62, 64). The most frequently used outcome was TUG, found in 14 studies (9, 30–32, 36, 40, 45, 50, 53, 55, 59, 62, 64, 69). Six studies combined TUG with the Tinetti gait score (9, 30, 31, 36, 40, 64). Four studies assessed gait speed using the 10MWT (32, 45, 59, 69). Three studies assessed functional performance with the 6MWT (44, 50, 56). Two studies used the Locometrix system for biomechanical analysis (31, 36).

##### Comparisons to control groups

Four meta analyses (9, 31, 36, 55) were conducted for the following outcomes: TUG test, 10MWT, Tinetti test and 6MWT.

**Figure 2:**
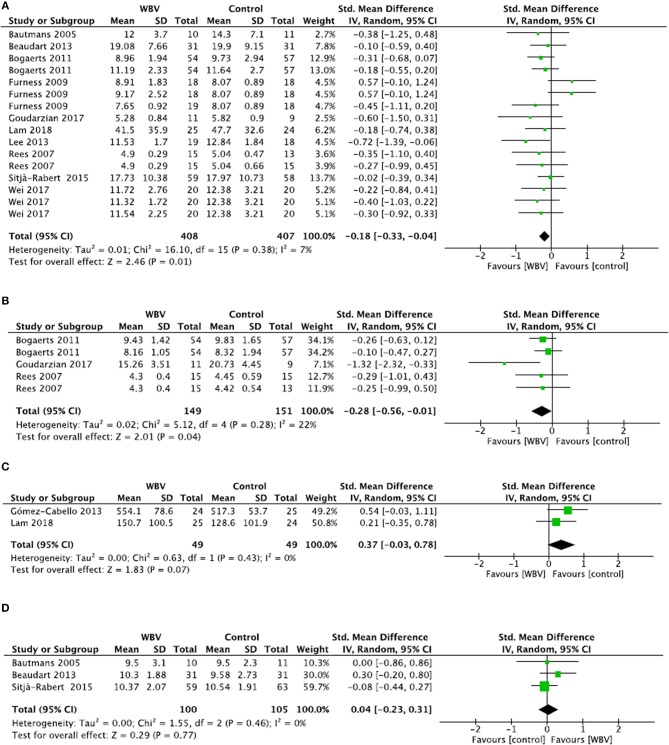
Comparison between WBV interventions and control groups in elderly subjects for the TUG test **(A)**, 10MWT **(B)**, 6MWT **(C)** and Tinetti gait score **(D)**.

For the TUG test (Figure 2A), 10 studies were included in meta-analysis and four studies were excluded due to a lack of data despite requests to the authors (9, 31, 36, 55). Meta-analysis showed a significant decrease in time in favor of the WBV groups (SMD = −0.18; 95% CI: −0.33, −0.04), with consistent results (*I*^2^ = 7%, *p* = 0.38). The included studies were of high quality (mean PEDro score = 5.8 ± 1.0), so a strong level of evidence supports the positive effect of WBV training on the TUG test.

For the 10MWT (Figure 2B), three studies were included in meta-analysis and one study was excluded because it used a different unit of measure (i.e., m/s instead of seconds in the other studies) (69). Meta-analysis showed a significant decrease in time on the 10MWT in WBV groups (SMD = −0.28; 95% CI: −0.56, −0.01), with consistent results (*I*^2^ = 22%, *p* = 0.28). The overall quality of the included studies was high (PEDro score = 5.0 ± 0.0). Thus, a strong level of evidence supports the positive effect of WBV training in improving gait speed on the 10MWT.

For the 6MWT (Figure 2C), two studies were included and one was excluded due to a lack of data despite requests to the authors (56). Meta-analysis showed no significant difference between groups (SMD = 0.37; 95% CI: −0.03, 0.78), despite a tendency toward an improvement in distance in WBV groups. Results were consistent (*I*^2^ = 0%, *p* = 0.43) and the quality of the included studies was high (PEDro score = 5.5 ± 0.7). Thus, a strong level of evidence supports the lack of a beneficial effect of WBV training for improving performance in the 6MWT.

For the Tinetti gait score (Figure 2D), three studies were included in meta-analysis and three were excluded due to a lack of data despite requests to the authors (9, 31, 36). Meta-analysis showed no significant difference between groups (SMD = 0.04; 95% CI: −0.23, 0.31), with consistent results (*I*^2^ = 0%, *p* = 0.46). The quality of the included studies was high (mean PEDro score = 7.0 ± 1.0). Thus, a strong level of evidence supports the absence of a positive effect of WBV training on the Tinetti gait score.

For biomechanical data recorded using the Locometrix system (gait speed, stride frequency, stride length, stride symmetry, stride regularity, cranio-caudal mechanic power, antero-posterior mechanic power, medio-lateral mechanic power, and counting speed), no comparison between groups could be performed due to a lack of data despite requests to the authors (31, 36). Both Beaudart et al. (31) and Buckinx et al. (36) reported no significant inter-group difference for parameters recorded by the Locometrix (*p* > 0.05).

#### Chronic COPD Patients

Four studies examined the effect of WBV on chronic COPD patients (57, 61, 65, 71) with an average PEDro score of 5.2 ± 0.5. The sample size ranged from 28 to 62 participants with a mean age of 66.2 ± 4.3 years. Three studies included both men and women (61, 65, 71) and one included only male patients (57). All of the studies specified the eligibility criteria and had similar baselines. The training duration varied from 6 weeks to 3 months. In two studies (57, 61), subjects performed three WBV sessions per week, while patients had only two sessions per week in the other two studies (65, 71). The frequency and amplitude of the platform vibrations varied from 6 to 35 Hz and 2 to 6 mm, respectively. Intensity was progressively increased in two studies (65, 71). Half of the studies used side-alternating vibrations (65, 71) while the two other studies used synchronous vibrations (57, 61). The number of vibration bouts delivered per session varied from three to eight sets with a period lasting between 30 s and 2 min for each. Resting time was 60 s to 2 min. In two protocols (57, 71), the subjects maintained a static position, while they performed dynamic exercises in the other studies (61, 65). Only the 6MWT methodology was used to test gait.

##### Comparisons to control groups (Figure 3A)

For the meta-analysis, two studies were included and two were excluded because the control groups were intervention groups with additional exercises not provided in the WBV group (i.e., not only WBV effects are measured) (61, 65). Meta-analysis showed no significant difference between groups (SMD = 1.66; 95% CI: −0.17, 3.49) with heterogeneous results (*I*^2^ = 91%, *p* = 0.0008). Thus, the level of evidence was conflicting for the 6MWT outcome in COPD.

**Figure 3:**
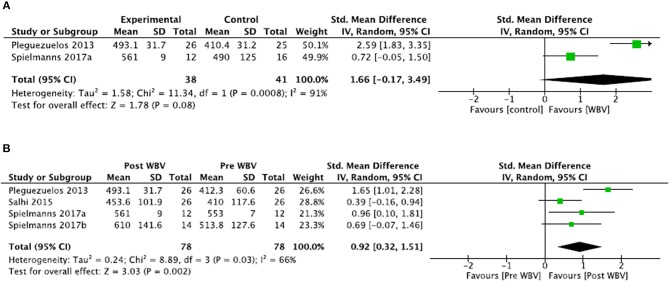
Comparisons between WBV interventions and control groups in COPD for the 6MWT **(A)**. Illustrates the change in the 6MWT following WBV intervention compared to the pre-intervention status **(B)**.

For the excluded studies, Salhi et al. (61) showed that there was no significant difference between WBV training and conventional resistance training for improving 6MWT scores (SMD = −0.24; 95% CI: −0.79, 0.31). Similar results were found by Spielmanns et al. (65), where no significant difference was shown between the WBV intervention and the calisthenics group (SMD = 0.54; 95% CI: −0.23, 1.32).

##### Comparison to pre-intervention (Figure 3B)

A second meta-analysis was conducted to include the four studies. Meta-analysis demonstrated a significant improvement in the distance walked during the 6MWT after WBV treatment (SMD = 0.92; 95% CI: 0.32, 1.51). Again, because there were heterogeneous results (*I*^2^ = 66%, *p* = 0.03), the level of evidence was conflicting for the 6MWT outcome.

#### Stroke Patients

Seven studies examined the effect of WBV on stroke patients (28, 35, 38, 51, 54, 67, 72) with an average PEDro score of 7.2 ± 1.7. The sample size ranged from 21 to 84 participants with a mean age of 58.3 ± 4.5 years. All of the studies included both men and women. All explained the eligibility criteria. Two studies (35, 38, 51, 72) found significant differences between groups for some outcomes at baseline. The training duration varied from 4 to 8 weeks. In four studies, subjects performed three sessions per week (28, 51, 54, 72), while patients had five sessions per week in two studies (38, 67), and two sessions per week in one study (35). The frequency and amplitude of platform vibrations varied from 20 to 40 Hz and 0.44 to 5 mm, respectively. The intensity was progressively increased in two studies (51, 72). Three studies used side-alternating vibrations (38, 67, 72), three synchronous vibrations (35, 51, 54), while one did not mention the type of vibrations (28). The number of vibration bouts delivered per session varied from 2 to 135 sets with a period lasting from 10 to 150 s each. Resting time was between 3 and 60 s. In four protocols (28, 35, 38, 67), the subjects maintained a static position, performed dynamic exercises in two studies (51, 72) and both types of exercises in one study (54). The TUG test was assessed in three studies (35, 38, 54), the 6MWT in three studies (35, 51, 54) and the 10MWT in two (28, 51). Only one study used a biomechanical methodology to assess gait function (72) and one study used the Functional Ambulation Categories (FAC) scale (67).

##### Comparisons to control groups (Figures 4A and 4C)

Two meta-analyses were conducted for the TUG test and the 6MWT.

**Figure 4:**
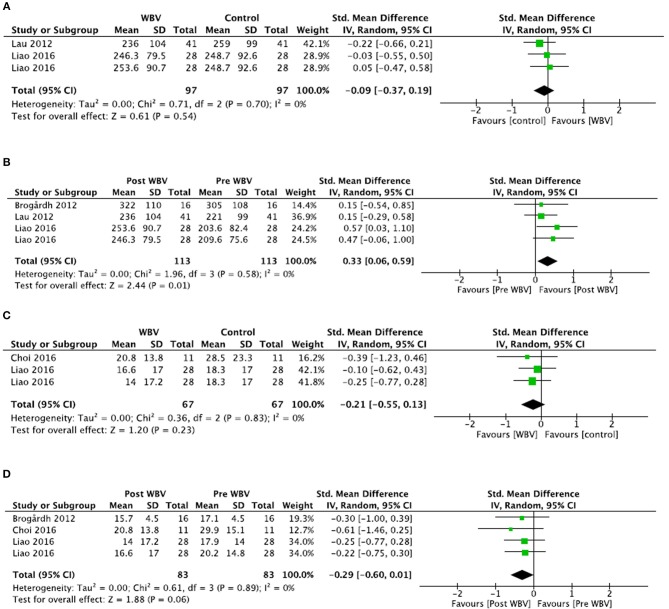
Comparisons between WBV interventions and control groups in stroke patients for the 6MWT **(A)** and the TUG test **(C)**. Illustrates the changes in the 6MWT **(B)** and the TUG test **(D)** following WBV intervention compared to the pre-intervention status.

For the TUG test, two studies were included and one study was excluded because the groups were statistically different at baseline (35). Meta-analysis demonstrated no significant difference between groups (SMD = −0.21; 95% CI: −0.55, 0.13), with consistent results (*I*^2^ = 0%, *p* = 0.83). The quality of the study was high (mean PEDro score = 8.0 ± 0.0). Thus, a strong level of evidence supports the absence of effect of WBV training on the TUG test in stroke patients.

For the 6MWT, two studies were included and one study was excluded because the groups were statistically different at baseline (35). Meta-analysis demonstrated no significant difference between the groups (SMD = −0.09; 95% CI: −0.37, 0.19), with consistent results (*I*^2^ = 0%, *p* = 0.70). The quality of the study was high (mean PEDro score = 6.0 ± 2.8). Thus, a strong level of evidence supports the absence of effect of WBV training on the 6MWT test in stroke patients.

For biomechanical data, Choi et al. (72) demonstrated no significant difference between groups for stride length (SMD = 0.50; 95% CI: −0.23, 1.23) and walking speed (SMD = 0.32; 95% CI: −0.40, 1.04). Similarly, walking speed assessed by the 10MWT (51) was not different between groups (SMD = 0.39; 95% CI: −0.05, 0.83). Finally, the Functional Ambulation categories scale (67) was not different between groups (SMD = 0.00; 95% CI: −0.54, 0.54) after the interventions. All studies were of high quality RCT (Perdro scores ≥ 5/10). Thus, the level of evidence for each outcome was considered moderate.

##### Comparisons to pre-intervention (Figures 4B and 4D)

Two additional meta-analyses were conducted to include the two studies excluded for group comparisons for the TUG test and the 6MWT outcomes.

For the TUG test, meta-analysis showed a tendency but no significant improvement after the WBV treatment (SMD = −0.29; 95% CI: −0.60, 0.01) with consistent results (*I*^2^ = 0%, *p* = 0.89). The overall quality of the included studies was high (mean PEDro score = 7.0 ± 2.6). Thus, a strong level of evidence supports the absence of effect of WBV treatment on the TUG test in stoke patients.

For the 6MWT, meta-analysis showed a significant improvement after WBV treatment (SMD = −0.33; 95% CI: 0.06, 0.59) with consistent results (*I*^2^ = 0%, *p* = 0.58). The overall quality of the included studies was high (mean PEDro score = 8.3 ± 0.5). Thus, a strong level of evidence supports the positive effect of WBV treatment to improve the distance walked during the 6MWT test in stroke patients.

#### Knee Osteoarthritis

Four studies examined the effect of WBV on patients suffering from knee osteoarthritis (29, 33, 63, 68). The studies had an average PEDro score of 6.5 ± 1.2. The sample size ranged from 21 to 49 subjects with a mean age of 65.1 ± 9.2 years. Two studies included both men and women (33, 68), while two studies did not mention the gender of the patients (29, 63). All of the studies specified the eligibility criteria and had similar baselines. The training duration ranged from 8 to 24 weeks. Three studies had a frequency of three sessions per week (29, 33, 63) while the other had five (68). The frequency and amplitude of the platform vibrations varied from 25 to 40 Hz and 2 to 6 mm, respectively. The intensity was progressively increased in all studies. Two studies used synchronous vibrations (33, 63) while two did not mention the type of vibrations of the devices (29, 68). The number of vibration bouts delivered per session varied from six to 30 sets with a period lasting 20 to 70 s. Resting time was between 20 and 70 seconds. In three protocols (29, 33, 68), the subjects maintained a static position, but performed static and dynamic exercises in the other study (63). Three studies used the TUG test (29, 33, 68), three used the 6MWT (29, 63, 68) and one combined the 2MWT and the 50FWT with the TUG (33).

##### Comparisons to control groups

**Figure 5:**
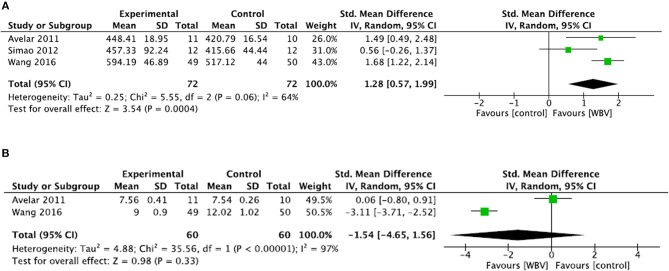
Comparison between WBV interventions and control groups in patients with knee OA for the 6MWT **(A)** and the TUG test **(B)**.

For the TUG test (Figure 5B), two studies were included and one was excluded due to a lack of data (33). Meta-analysis showed no significant difference between groups (SMD = −1.54; 95% CI: −4.65, 1.56) with heterogeneous results (*I*^2^ = 97%, *p* < 0.00001). Thus, the level of evidence was conflicting.

For the 6MWT (Figure 5A), meta-analysis showed a significant difference in favor of the WBV group (SMD = 1.28; 95% CI: 0.57, 1.99), with consistent results (*I*^2^ = 64%, *p* = 0.06). The quality of the studies was high (mean PEDro score = 6.6 ± 1.5). Thus, a strong level of evidence supports the positive effect of adding WBV to improve the 6MWT in patients with knee OA.

#### Postmenopausal Women

Three studies examined the effect of WBV on postmenopausal patients (48, 58, 66). These studies had an average PEDro score of 4.6 ± 1.1. The sample size ranged from 27 to 52 participants with a mean age of 65.8 ± 8.4 years. All of the studies specified eligibility and had similar baselines. The training durations ranged from 4 weeks to 8 months. One study had a frequency of five sessions per week (66), another of three sessions per week (58), while the last one did not specify the number of sessions per week (48). The frequency of the platform vibrations varied from 6 to 35 Hz and the amplitude was indicated in only one study (6 mm). The intensity of the sessions was progressively increased in two studies during training duration (58, 66). One study used synchronous vibrations (66) and the other two studies did not mention the type of vibrations (48, 58). The vibration bouts were delivered from 30 to 60 s with two to six sets. Resting time was 60 s in two studies (58, 66) and was not indicated in the third (48). In all protocols, the subjects maintained a static standing position. Two studies used the TUG (48, 66) and one combined it with a 10MWT (48). The third study measured walking speed along a four-meter pathway (58). Meta-analysis could not be performed for the TUG test due to a lack of post-intervention data in all three studies, despite requests to the authors. Two studies reported significant improvement of the 10MWT after WBV training (*p* < 0.05 and *p* = 0.006) (48, 58). Sucuoglu et al. (66) showed a significant improvement of the TUG test post treatment (*p* < 0.005), whereas Iwamoto et al. (48) found no significant difference between groups (*p* > 0.05).

#### Multiples Sclerosis

Two studies examined the effect of WBV on patients with multiple sclerosis (34, 39). The studies had an average PEDro score of 6.0 ± 1.4. The sample size ranged from 25 to 34 participants with a mean age of 43.4 ± 6.3 years. Both studies included both men and women, specified the eligibility criteria and had similar baselines. The training duration was 10 and 20 weeks. In one study, patients underwent three sessions per week (39), while in the other they performed an average of 2.5 sessions per week (34). The frequency and amplitude of the platform vibrations varied from 2 to 45 Hz and 2 to 2.5 mm, respectively. The intensity was progressively increased in both studies. One study used synchronous vibrations (34) while the other did not mention the type of vibrations (39). The number of vibration bouts delivered per session varied from 2 to 15 sets with a period lasting 30 to 120 s. Resting time was between 30 and 120 s. In one protocol (39), the subjects maintained a static position, while they performed static and dynamic exercises in the other (34). Both studies used the TUG test. One study combined it with the 10MWT and the 6MWT (39), while the other used the TUG with the 2MWT and the 25-foot walk test (34).

##### Comparisons to control groups

In one study, no meta-analysis was conducted for between-group comparisons because the groups were statistically different at baseline for the TUG test and 2MWT (34).

Ebrahimi et al. (39) found no significant difference between groups for the TUG test (SMD = −0.47; 95% CI: −1.20, 0.26). However, they did observe significant improvement in the WBV group for the 10MWT (SMD = −1.05; 95% CI: −1.82, −0.28) and the 6MWT (SMD = 1.22; 95% CI: 0.43, 2.01). The level of evidence was high (PEDro score = 5/10). Thus, the level of evidence was considered moderate for each outcome.

##### Comparison to pre-intervention

**Figure 6:**
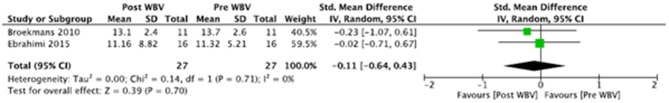
Changes in the TUG test following WBV intervention compared to the pre-intervention status in patients with multiple sclerosis.

Meta-analysis (Figure 6) showed no significant improvement in the TUG test after WBV training (SMD = −0.11; 95% CI−0.64, 0.43) with consistent results (*I*^2^ = 0%, *p* = 0.71). The overall quality of the included studies was high (mean PEDro score = 6.0 ± 1.4). Thus, there is a strong level of evidence to conclude that WBV treatment had no impact on the TUG test in patients with multiple sclerosis.

#### Other Pathologies in Adults

Six studies reported results on different pathologies in adult patients(27, 41–43, 47, 49): incomplete cervical spinal injury (47), pulmonary arterial hypertension (42), lung transplantation (43), idiopathic Parkinson's disease (41), total knee arthroplasty (49) and cerebral palsy (27). The average PEDro score was 5.3 ± 1.7. The sample size ranged from 14 to 83 subjects with a mean age of 54.6 ± 14.1 years. All of the studies included both men and women. All specified the eligibility criteria and had similar baselines except for one study where patients were statically different at baseline for certain outcomes (49). Training duration varied from 4 to 8 weeks. In four studies, subjects performed three WBV sessions per week (27, 41, 43, 49), while in one study they had four sessions per week (42), and five in another (47). The frequency and amplitude of the platform vibrations varied from 6 to 40 Hz and 2 to 20 mm, respectively. The frequency or amplitude of the vibrations was progressively increased in four studies (27, 43, 47, 49). Two studies used side-alternating vibrations (42, 43), one study used synchronous vibrations (47) and the other three studies did not mention the type of vibrations (27, 41, 49). The number of vibration bouts delivered per session varied from 1 to 18 sets with a period lasting 30 to 120 s. Resting time was between 15 and 240 s. In three protocols (27, 41, 47), the subjects maintained a static position, while they performed dynamic exercises in two others (42, 43) and combined both in the last study (49). Four studies used the TUG test (27, 41, 47, 49) and three used the 6MWT (27, 42, 43) (one study combined both). One study used biomechanical analysis combined with the clinical TUG test (41). No meta-analysis was conducted due to the heterogeneity of patients in this subgroup.

##### Comparisons to control groups

In patients with incomplete cervical spinal injury, In et al. (47) found no significant difference between the WBV group and control group for the TUG test (SMD = −0.64; 95 CI: −1.40, 0.13) and the 10MWT (SMD = −0.23; 95% CI: −0.97, 0.52). The quality of the study was high (PEDro score = 8/10). Thus, the level of evidence was moderate.

In patients diagnosed with idiopathic Parkinson's disease, Gaßner et al. (41) observed no significant difference between the WBV group and the placebo group for the TUG test (SMD = −0.37; 95% CI: −1.34, 0.59), gait velocity (SMD = −0.21; 95% CI: −1.17, 0.74) and step length (SMD = 0.14; 95% CI: −0.81, 1.09). The quality of the study was high (PEDro score = 5/10). Thus, the level of evidence was moderate.

After total knee arthroplasty, Johnson et al. (49) reported no significant difference for the TUG test between a WBV group and a resistance training group (SMD = −0.59; 95% CI: −1.59, 0.42). The quality of the study was low (PEDro score = 3/10). Thus, the level of evidence was limited.

In patients with cerebral palsy, Ahlborg et al. (27) found no significant difference between a WBV group and a resistance training group for the TUG test (SMD = 0.28; 95% CI: −0.77, 1.34). The quality of the study was high (PEDro score = 6/10). Thus, the level of evidence was moderate.

SMD could not be reported in the study of Gerhardt et al. (42) and Gloeckl et al. (43) due to the lack of post-intervention data despite a request to the authors. The authors of the first study indicated that WBV was associated with a significant improvement of the 6MWD vs. baseline of +38.6 ± 6.6 m (*p* < 0.001) (42). The authors of the second study reported a significant between-group difference of 28 m (95% CI: 3, 54; *p* = 0.029) in favor of WBV (73).

##### Comparisons to pre-intervention

Ahlborg et al. (27) reported no significant difference after WBV (SMD = 0.14; 95% CI: −0.91, 1.19) in patients with cerebral palsy, with a level of evidence considered moderate (PEDro score = 6/10). Similarly, Johnson et al. (49) observed no significant improvement of the TUG test after WBV (SMD = 1.02; 95% CI: −0.04, 2.09) in patients with total knee arthroplasty, with a limited level of evidence (PEDro score = 3/10).

#### Other Pathologies in Children

Four studies examined the effect of WBV in children(37, 46, 52, 60): two evaluated the effects of WBV in cerebral palsy (37, 60), one in patients with osteogenesis imperfect (46) and one in patients with spastic diplegia or quadriplegia forms of cerebral palsy (52). The studies had an average PEDro score of 6.0 ± 1.4. The sample size ranged from 16 to 30 participants with a mean age of 8.7 ± 0.8 years. All of the studies included both boys and girls, specified the eligibility criteria and had similar baselines. The training duration varied between 8 and 24 weeks. One study had a frequency of two sessions per week (46), one had three sessions per week (52), one had five sessions per week (60), while the number of sessions was not specified in the last study (37). The frequency and amplitude of the platform vibrations varied from 5 to 25 Hz and 1 to 9 mm, respectively. The frequency or amplitude of the vibrations was progressively increased in three studies (46, 52, 60). Two studies used synchronous vibrations (37, 60) and the other two studies used side-alternating vibrations (46, 52). The number of vibration bouts delivered per session varied from three to six sets with periods lasting 3 min each. Resting time was also 3 min. In two protocols (37, 52), the subjects maintained a static position, while they performed static and dynamic exercises in the other studies(46, 60). Two studies used the 6MWT (37, 46), and one study combined it with the TUG test (37). One study used biomechanical analysis including gait speed, stride length and cycle time (52). One study used the 10MWT (for gait speed) (60). Three studies reported significant improvement in gait parameters following WBV treatment (37, 52, 60), whereas one study reported that the 6MWT remained unchanged (46). No meta-analysis was conducted due to the heterogeneity of patients in this subgroup. Additionally, data post interventions were not reported in three studies (37, 46, 60).

##### Comparisons to control groups

In children with clinically mild to moderate osteogenesis imperfecta, Högler et al. reported no significant difference between groups for the 6MWT (*p* = 0.278) (46).

In children with cerebral palsy, Cheng et al. reported a significant difference between the treatment and control conditions for the 6MWT (*p* = 0.005) (37). Ruck et al. reported a significant improvement in the 10MWT in favor of WBV (*p* = 0.03) (60).

Finally, in patients with either spastic diplegia or quadriplegia forms of cerebral palsy, Lee and Chon found significant improvement in favor of the WBV group for gait speed (SMD = 1.41; 95% CI: 0.60, 2.22) and stride length (SMD = 0.91; 95% CI: 0.15, 1.67) (52), with a level of evidence considered moderate (PEDro score = 8/10).

## Discussion

The aim of this systematic review was to determine the changes in gait outcomes after WBV training in healthy adults and various patient categories. We found a strong level of evidence for a positive effect of WBV training on the TUG test and the 10MWT in the elderly. The same level of evidence was found in favor of a significant improvement of the 6MWT in stroke patients and patients with knee OA. In contrast, there is no change in the 6MWT and the Tinetti gait score in the elderly, and the TUG test was not improved in stroke or multiple sclerosis patients. Conflicting results were found in COPD patients despite significant improvements in the 6MWT. Other outcomes showed a moderate or limited level of evidence, due to a lack of data or because only one RCT was identified.

As mentioned in a prior review (20), the major obstacle in conducting meta-analysis and establishing strong evidence on the effects of WBV on gait is the heterogeneity in study methodologies. Intervention regimes, settings, combined interventions and control groups varied greatly. As in any training protocol, numerous factors can affect the results of the program (e.g., the duration of the intervention; the frequency or volume of the sessions; the type, frequency and amplitude of the vibrations and the exercises performed on the platform). Because the studies used different protocols, a random-effects model was used. In the presence of heterogeneity, a random-effects meta-analysis weights the studies relatively more equally than a fixed-effect analysis (25). Because control groups varied a great deal in terms of interventions (i.e., exercises, physical therapy, sham, no interventions etc.), intergroup comparisons were not always possible, so we added within group comparisons (i.e., pre vs. post WB) to estimate the effect of the treatment. Additionally, some groups were statistically different at baseline for certain outcomes (35, 40, 62), making between groups comparisons impossible after intervention. The results of each patient category are discussed below.

On the one hand, the results in the elderly showed significant improvements in the TUG test and the 10MWT after WBV intervention. These results are in favor of better dynamic stability and gait performance as both outcomes are related to balance and gait speed, respectively (45, 59).

However, the effect sizes were small (−0.18 and−0.28, respectively) and the 6MWT was not significantly modified by treatment despite a tendency toward improvement in favor of WBV (SMD = 0.37; 95% CI: −0.03, 0.78). Our findings on physical improvements partially corroborate earlier reports. In a recent scoping review, Park et al. (74) concluded that WBV training could be effective in increasing lean mass, muscular strength and cardiovascular health (74). Positive changes in body composition and fitness induced by WBV training may explain the improvement in gait performance. After the age of 50, muscle mass decreases approximately 2% every year and muscle strength decreases 15% every 10 years (75). These age-related changes impact functional mobility, including gait speed, static dynamic balance, and the risk of falling. As a resistance training exercise, WBV appears efficient in attenuating the loss of muscle mass and muscle strength. In order to combat the effects of aging, it should be recommended that older adults perform WBV 2 or 3 days per week, as suggested for resistance training (76, 77). In addition, because both offer numerous benefits, these interventions could be combined, depending on feasibility and patient motivation. These recommendations are also valuable for patients with reduced mobility and who need to improve their autonomy at home, as only the TUG test and 10MWT (short-distance walking tests) were improved but not the 6MWT (a long-distance walking test).

On the other hand, the results support that gait performance can be improved with no improvement in qualitative aspects of the locomotion pattern. In fact, the quality of gait, assessed by the Tinetti test, was not changed. The outcome is classically divided in two parts. One assesses static balance, while the second asses dynamic balance (78, 89). Because gait was the main outcome of the present research, we did not include the total score in meta-analysis. All 28 points are necessary to assess the whole balance score, and thus, the risk of falling. Additionally, the Tinetti total score has been recently demonstrated as being related to muscle mass and strength (79). As previously discussed, improvements in muscle mass, strength, and performance are demonstrated after WBV training. Thus, changes in the Tinetti total score can be expected and further investigation on this outcome is warranted.

The results in stroke patients are more mitigated. On the one hand, between-group comparisons showed that the 6MWT was not modified by WBV training. However, two studies had to be excluded from this analysis and a second analysis was performed to include them in a pre vs. post comparison. This time, the results showed a significant increase in distance after a WBV intervention. On the other hand, no significant changes were found for the TUG test in both comparisons, despite a tendency toward an improvement. The 6MWT is commonly used to assess aspects of walking performance in stroke survivor studies (80). It evaluates the global responses of all the systems involved during exercise, including the pulmonary and cardiovascular systems, systemic circulation, peripheral circulation, blood, neuromuscular units, and muscle metabolism (90). The results concerning the 6MWT confirm the findings discussed previously in favor of functional improvements in elderly and disease populations (74). Conflicting results emerged from the TUG test. Balance, assessed by the TUG test, appeared less modified by the WBV treatment in the treatment of neuropathologic subjects than in the elderly population. This was confirmed by the poor results in multiple sclerosis patients included in meta-analysis for the TUG outcome (34, 39).

Conflicting results were also found for the 6MWT in COPD patients. The pooled studies demonstrated a significant improvement of the distance walked but with inconsistent results. The size of the overall effect was large (SMD = 0.92; 95% CI:0.32, 1.51) and seems to corroborate the functional improvement observed after WBV. However, the results must be taken with caution because of their heterogeneity and the small sample of high quality studies revealed by the present review (i.e., four studies) (57, 61, 65, 71). Heterogeneity was found in both intergroup comparison and comparison with pre intervention, and was difficult to explain as the studies had similar populations (i.e., older adults with COPD) and settings (i.e., frequency and amplitude of the vibrations).

In patients with knee OA, a strong level of evidence supports the beneficial effect of WBV training in improving the 6MWT, with a large effect size (SMD = 1.28; 95% CI: 0.57, 1.99). Interestingly, Wang et al. showed that adding WBV to quadriceps resistance training was more efficient that resistance training alone (SMD = 1.68; 95% CI: 1.22, 2.14) (68). A recent review showed that, in patients with knee OA, a resistance training program is effective for improving knee extensor strength but has limited effect on pain and disability if the gains are <30% (81). WBV interventions combined with strength training may help achieve this gain necessary for beneficial effects on pain and functional performance. Again, for the TUG test, Wang et al. (68) demonstrated the value of combining WBV and resistance training to improve gait performance (SMD = −3.11; 95% CI: −3.71, −2.52). However, because of the absence of effect in the second study (29), the level of evidence was conflicting. Heterogeneity might be explained by the two major differences between the studies, which were the addition of quadriceps resistance training and the doubled duration of the training (i.e., 24 vs. 12 weeks) in Wang et al. (68).

Although some significant gait improvements after WBV were reported, it is important to stress that significant statistical changes are not always linked to significant clinical improvements for patients. For example, in elderly patients, Bogaerts et al. (32) reported a significant improvement of the time required to perform the TUG test after WBV intervention. While the time was decreased from 13.1 s to 11.19 s (SMD = −0.71; 95%CI: −1.10, −0.32) with an effect size considered moderate, this difference may not correspond to major changes in the patients' daily activities. Thus, the benefit of a non-functional intervention such as WBV should always be questioned with regard to each patient's goals.

However, WBV appears to be a time-efficient and easy-to-use intervention that is both relatively inexpensive and safe for patients with balance deficits. Vibration plates are readily available at all rehabilitation hospitals/centers. Moreover, it might be interesting to complete certain conventional rehabilitation programs like resistance or balance training with WBV training that may offer the same results. For example, for COPD patients, it has been found that both the WBV and resistance training groups significantly improved in the 6MWT (61, 65, 71) with no significant difference between the groups. Additionally, no control group including any form of training was significantly superior to WBV training in improving gait.

Most of the studies included in this review (27/43 articles) reported a drop-out rate of <15% during their interventions. Considering that most of the subjects were patients with diseases or physical disorders, it is logical to assume that they would have stopped treatment had they experienced any harmful or adverse side effect. This might support the hypothesis that the patients tolerated vibration training well. Moreover, WBV training has been reported to be appreciated and considered a safe training method. The fact that participants could perform either dynamic or static exercises while holding a bar increases safety and would be beneficial for the weakest populations such as elderly persons with balance impairments.

The vibration type may impact the training response. side-alternating WBV has been shown to increase heart rate higher than synchronous vibrations in young sedentary women during 20-min sessions (82). This illustrates the potential of WBV to improve fitness capacity, particularly in less active populations. Additionally, higher electromyography of knee extensor and plantar flexor activities were observed with a side-alternating vibration platform compared to synchronous vibrations (83). Although these results are in favor of side-alternating vibrations, our review showed heterogeneous results regarding the vibration type when it was mentioned. Significant improvements in gait parameters were found in 13 studies that used synchronous vibrations (9, 30, 33, 37, 40, 47, 51, 57, 59–61, 63, 66) and in seven studies that used side-alternating vibrations (38, 42, 43, 48, 52, 65, 72). Again, because of the lack of consistency in the protocols and results, it is difficult to reach a consensus on specific WBV training to improve human locomotion.

We chose to select only studies on long-term effects because they are better correlated to conventional physiotherapies that often last many weeks. Moreover, long-term effects have been studied more than short-term or even immediate effects. We found that a wide range of protocols lasted 6 weeks or more and a few lasted 4 weeks. However, we can add that only a few RCT focused on the acute effects of WBV training on gait parameters (84–86). In the future, it might be interesting to compare different WBV protocols (i.e., with different WBV frequencies) in order to evaluate the effect of high vs. low WBV frequency on balance and gait within a single session.

Finally, most studies used clinical assessments instead of biomechanical analysis (41, 52, 72). Since it might be a more objective measure, future studies should integrate this kind of outcome more often in order to compare it with functional assessments.

## Limitations of the Study

Results of meta-analysis must be taken with caution as some studies could not be included in the comparisons due to a lack of complete data, notably for the TUG test and Tinetti gait score in the elderly, despite requests to the authors.

The Cochrane Qualitative and Implementation Methods Group recommends the application of Grades of Recommendation, Assessment, Development, and Evaluation (GRADE) in the Evidence from Qualitative Reviews to assess confidence in qualitative synthesized findings (87). However, the GRADE necessitates assessing the risk of publication bias with a funnel plot, determining its asymmetry, which can be performed with at least ten studies (88). Because most of the statistical analyses were conducted on few RCT, we decided to implement other guidelines described by a Cochrane collaboration group to assess the level of evidence (26). Because this method includes fewer criteria, our confidence in the results must be taken with caution.

## Conclusion

While WBV training appears to be a useful and relatively successful tool in improving gait and walking abilities, it remains unclear whether the treatment could be generalized to all patients. Some populations have been studied more than others with varying degrees of consistency. In the elderly, there is a strong level of evidence that WBV can improve mobility by improving the TUG test, and gait speed by improving the 10MWT. The results also showed significant improvements to functional performance in stroke patients and patients with knee OA by improving the 6MWT. However, the treatment was inefficient in changing the TUG test in stroke and multiple sclerosis patients, and conflicting results were obtained for the 6MWT in COPD. Finally, other outcomes were studied less and the level of evidence was moderate or even limited depending of the quality of the study. The transferability of this kind of training to daily activities remains unclear and the use of vibration training to replace functional rehabilitation must always be questioned. Further research is needed to explore the possibility of finding a standardized protocol targeting gait ability in a wide range of populations.

## Author Contributions

MF, TV, GL, PF, TH, LC, J-LH, EY, P-AD, and AD designed the study, collected, analyzed, and interpreted the data, drafted and revised the manuscript, tables and figures, and gave final approval.

### Conflict of Interest Statement

The authors declare that the research was conducted in the absence of any commercial or financial relationships that could be construed as a potential conflict of interest.

## References

[1] Goetz CG. Jean-martin charcot and his vibratorychair for Parkinson disease. Neurology. (2009) 73:475–8. 10.1212/WNL.0b013e3181b1640b 19667323

[2] Kaeding TS Sarkopenie und vibrationstraining : eine übersicht. Zeitschrift fur Gerontologie Geriatrie. (2009) 42:88–92. 10.1007/s00391-008-0565-4 18726053

[3] Weber-Rajek M Mieszkowski J Niespodzinski B Ciechanowska K. Whole-body vibration exercise in postmenopausal osteoporosis. Prz Menopauzalny. (2015) 14:41–7. 10.5114/pm.2015.48679 26327887 PMC4440196

[4] Wang XQ Pi YL Chen PJ Chen BL Liang LC Li X. Whole body vibration exercise for chronic low back pain: study protocol for a single-blind randomized controlled trial. Trials. (2014) 15:104. 10.1186/1745-6215-15-104 24693945 PMC4230279

[5] Collado-Mateo D Adsuar JC Olivares PR del Pozo-Cruz B Parraca JA del Pozo-Cruz J. Effects of whole-body vibration therapy in patients with fibromyalgia: a systematic literature review. Evid Based Compl Alt Med. (2015) 2015:719082. 10.1155/2015/719082 26351517 PMC4553315

[6] Cochrane DJ. The potential neural mechanisms of acute indirect vibration. J Sports Sci Med. (2011) 10:19–30. 24149291 PMC3737901

[7] Aminian-Far A Hadian MR Olyaei G Talebian S Bakhtiary AH. Whole-body vibration and the prevention and treatment of delayed-onset muscle soreness. Journal of Athletic Training. (2011) 46, 43–49. 10.4085/1062-6050-46.1.43 21214349 PMC3017487

[8] Melnyk M Kofler B Faist M Hodapp M Collhofer A. Effect of a whole-body vibration session on knee stability. Int J Sports Med. (2008) 29:839–44. 10.1055/s-2008-1038405 18401809

[9] Bruyere O Wuidart M-A Palma ED Gourlay M Ethgen O Richy F. Controlled whole body vibration to decrease fall risk and improve health-related quality of life of nursing home residents. Arch Phys Med Rehabilit. (2005) 86:303–7. 10.1016/j.apmr.2004.05.019 15706558

[10] Zaidell LN Mileva KN Sumners DP Bowtell JL. Experimental evidence of the tonic vibration reflex during whole-body vibration of the loaded and unloaded leg. PLoS ONE. (2013) 8. 10.1371/journal.pone.0085247 24386466 PMC3875536

[11] Martin BJ Park HS. Analysis of the tonic vibration reflex: influence of vibration variables on motor unit synchronization and fatigue. Eur J Appl Physiol Occup Physiol. (1997) 75:504–11. 10.1007/s004210050196 9202946

[12] Mileva KN Bowtell JL Kossev AR. Effects of low-frequency whole-body vibration on motor-evoked potentials in healthy men. Exp Physiol. (2009) 94:103–16. 10.1113/expphysiol.2008.042689 18658234

[13] Games KE Sefton JM Wilson AE. Whole-body vibration and blood flow and muscle oxygenation: a meta-analysis. J Athl Train. (2015) 50:542–9. 10.4085/1062-6050-50.2.09 25974682 PMC4560014

[14] Rogan S Taeymans J Radlinger L Naepflin S Ruppen S Bruelhart Y. Effects of whole-body vibration on postural control in elderly: an update of a systematic review and meta analysis. Arch. Gerontol. Geriatr. (2017) 73:95–112. 10.1016/j.archger.2017.07.022. 28800481

[15] Takakusaki K. Functional neuroanatomy for posture and gait control. J Mov Disord. (2017) 10:1–17. 10.14802/jmd.16062 28122432 PMC5288669

[16] Breniere Y Do MC. When and how does steady state gait movement induced from upright posture begin? J Biomech. (1986) 19:1035–40. 3818673 10.1016/0021-9290(86)90120-x

[17] Brenière Y Do MC. Control of gait initiation. J Mot Behav. (1991) 23:235–40. 10.1080/00222895.1991.9942034 14766505

[18] Winter D Human balance and posture control during standing and walking. Gait Posture. (1995) 3:193–214. 10.1016/0966-6362(96)82849-9

[19] Hendrickson J Patterson KK Inness EL McIlroy WE Mansfield A. Relationship between asymmetry of quiet standing balance control and walking post-stroke. Gait Posture. (2014) 39:177–81. 10.1016/j.gaitpost.2013.06.022 23877032

[20] Lindberg J Carlsson J. The effects of whole-body vibration training on gait and walking ability - a systematic review comparing two quality indexes. Physiother Theory Pract. (2012) 28:1–14. 10.3109/09593985.2011.641670 22214345

[21] Natalie A de Morton. The PEDro scale is a valid measure of the methodological quality of clinical trials: a demographic study. Aust J Physiother. (2009) 55, 129–133. 10.1016/S0004-9514(09)70043-1 19463084

[22] Wan X Wang W Liu J Tong T Estimating the sample mean and standard deviation from the sample size, median, range and/or interquartile range. BMC Med Res Methodol. (2014) 14. 10.1186/1471-2288-14-135 PMC4383202 25524443

[23] RevMan 5 download (2019). Available online at: /help/tools-and-software/revman-5/revman-5-download (accessed March, 26 2019).

[24] Cohen J Statistical Power Analysis for the Behavioral Sciences, 2nd ed. (1988). Hillsdale, N.J: L. Erlbaum Associates.

[25] Higgins J Green S editors. Cochrane Handbook for Systematic Reviews of Interventions. Version 5.1.0. (2011) The Cochrane Collaboration Available online at: www.cochrane-handbook.org(accessed March 2011).

[26] van Tulder M Furlan A Bombardier C Bouter L. Updated method guidelines for systematic reviews in the cochrane collaboration back review group. Spine. (2003) 28, 1290–1299. 10.1097/01.BRS.0000065484.95996.AF 12811274

[27] Ahlborg L Andersson C Julin P. Whole-body vibration training compared with resistance training: effect on spasticity, muscle strength and motor performance in adults with cerebral palsy. J Rehabilit Med. (2006) 38:302–8. 10.1080/16501970600680262 16931460

[28] Alp A Efe B Adali M Bilgiç A Demir Türe S Coşkun S. The impact of whole body vibration therapy on spasticity and disability of the patients with poststroke hemiplegia. Rehabilit. Res. Prac. (2018) 2018, 1–6. 10.1155/2018/8637573 30225145 PMC6129331

[29] Avelar NCP Simão AP Tossige-Gomes R Neves CDC Rocha-Vieira E Coimbra CC. The effect of adding whole-body vibration to squat training on the functional performance and self-report of disease status in elderly patients with knee osteoarthritis: a randomized, controlled clinical study. J Alt Compl Med. (2011) 17:1149–55. 10.1089/acm.2010.0782 22087576

[30] Bautmans I Van Hees E Lemper JC Mets T. The feasibility of whole body vibration in institutionalised elderly persons and its influence on muscle performance, balance and mobility: a randomised controlled trial [ISRCTN62535013]. BMC Geriatr. (2005) 5. 10.1186/1471-2318-5-17 16372905 PMC1368976

[31] Beaudart C Maquet D Mannarino M Buckinx F Demonceau M Crielaard JM. Effects of 3 months of short sessions of controlled whole body vibrations on the risk of falls among nursing home residents. BMC Geriatr. (2013) 13:42. 10.1186/1471-2318-13-42 23647914 PMC3649886

[32] Bogaerts A Delecluse C Boonen S Claessens AL Milisen K Verschueren SMP. Changes in balance, functional performance and fall risk following whole body vibration training and vitamin D supplementation in institutionalized elderly women. A 6 month randomized controlled trial. Gait Posture. (2011) 33:466–472. 10.1016/j.gaitpost.2010.12.027 21256028

[33] Bokaeian HR Bakhtiary AH Mirmohammadkhani M Moghimi J. The effect of adding whole body vibration training to strengthening training in the treatment of knee osteoarthritis: a randomized clinical trial. J Bodyw Movement Therap. (2016) 20:334–40. 10.1016/j.jbmt.2015.08.005 27210851

[34] Broekmans T Roelants M Alders G Feys P Thijs H Eijnde B. Exploring the effects of a 20-week whole-body vibration training programme on leg muscle performance and function in persons with multiple sclerosis. J Rehabili Med. (2010) 42:866–72. 10.2340/16501977-0609 20878048

[35] Brogårdh C Flansbjer U-B Lexell J. No specific effect of whole-body vibration training in chronic stroke: a double-blind randomized controlled study. Arch Phy Med Rehabilit. (2012) 93:253–8. 10.1016/j.apmr.2011.09.005 22289234

[36] Buckinx F Beaudart C Maquet D Demonceau M Crielaard JM Reginster JY. Evaluation of the impact of 6-month training by whole body vibration on the risk of falls among nursing home residents, observed over a 12-month period: a single blind, randomized controlled trial. Aging Clin Exp Res. (2014) 26:369–76. 10.1007/s40520-014-0197-z 24469903

[37] Cheng HYK Yu YC Wong AMK Tsai YS Ju YY. Effects of an eight-week whole body vibration on lower extremity muscle tone and function in children with cerebral palsy. Res Dev Disabil. (2015) 38:256–61. 10.1016/j.ridd.2014.12.017 25575288

[38] Choi E-T Kim Y-N Cho W-S Lee D-K. The effects of visual control whole body vibration exercise on balance and gait function of stroke patients. J Phys Ther Sci. (2016) 28:3149–52. 10.1589/jpts.28.3149 27942138 PMC5140818

[39] Ebrahimi A Eftekhari E Etemadifar M. Effects of whole body vibration on hormonal & functional indices in patients with multiple sclerosis. Indian J Med Res. (2015) 142:450–8. 10.4103/0971-5916.169210 26609037 PMC4683830

[40] Furness TP Maschette WE. Influence of whole body vibration platform frequency on neuromuscular performance of community-dwelling older adults. J Strength Cond Res. (2009) 23:1508–13. 10.1519/JSC.0b013e3181a4e8f9 19620913

[41] Gaßner H Janzen A Schwirtz A Jansen P. Random whole body vibration over 5 weeks leads to effects similar to placebo: A controlled study *in* Parkinson's Disease. Parkinson's Dis. (2014) 2014:1–9. 10.1155/2014/386495 25371843 PMC4211146

[42] Gerhardt F Dumitrescu D Gärtner C Beccard R Viethen T Kramer T. Oscillatory whole-body vibration improves exercise capacity and physical performance in pulmonary arterial hypertension: a randomised clinical study. Heart. (2017) 103:592–8. 10.1136/heartjnl-2016-309852 28100544 PMC5529961

[43] Gloeckl R Heinzelmann I Seeberg S Damisch T Hitzl W Kenn K. Effects of complementary whole-body vibration training in patients after lung transplantation: a randomized, controlled trial. J Heart Lung Transpl. (2015) 34:1455–61. 10.1016/j.healun.2015.07.002 26279196

[44] Gómez-Cabello A González-Agüero A Ara I Casajús JA Vicente-Rodríguez G. Effects of a short-term whole body vibration intervention on physical fitness in elderly people. Maturitas. (2013) 74:276–8. 10.1016/j.maturitas.2012.12.008 23312489

[45] Goudarzian M Ghavi S Shariat A Shirvani H Rahimi M. Effects of whole body vibration training and mental training on mobility, neuromuscular performance, and muscle strength in older men. J Exer Rehabilit. (2017) 13:573–80. 10.12965/jer.1735024.512 29114533 PMC5667605

[46] Högler W Scott J Bishop N Arundel P Nightingale P Mughal MZ The effect of whole body vibration training on bone and muscle function in children with osteogenesis imperfecta. J Clin Endocrinol Metabol. (2017) 102:2734–43. 10.1210/jc.2017-00275 28472303

[47] In T Jung K Lee M-G Cho H. Whole-body vibration improves ankle spasticity, balance, and walking ability in individuals with incomplete cervical spinal cord injury. NeuroRehabilitation. (2018) 42:491–7. 10.3233/NRE-172333 29660953

[48] Iwamoto J Sato Y Takeda T Matsumoto H. Whole body vibration exercise improves body balance and walking velocity in postmenopausal osteoporotic women treated with alendronate: Galileo and Alendronate Intervention Trail (GAIT). J Musculoskelet Neuronal Interact. (2012) 12:136–43. 22947545

[49] Johnson AW Myrer JW Hunter I Feland JB Hopkins JT Draper DO. Whole-body vibration strengthening compared to traditional strengthening during physical therapy in individuals with total knee arthroplasty. Physiother Theor Prac. (2010) 26:215–25. 10.3109/09593980902967196 20397856

[50] Lam FM Chan PF Liao L Woo J Hui E Lai CW. Effects of whole-body vibration on balance and mobility in institutionalized older adults: a randomized controlled trial. Clin Rehabil. (2018) 32:462–72. 10.1177/0269215517733525 29019274

[51] Lau RWK Yip SP Pang MYC. Whole-body vibration has no effect on neuromotor function and falls in chronic stroke. Med Sci Sports Exerc. (2012) 44:1409–1418. 10.1249/MSS.0b013e31824e4f8c 22330025

[52] Lee B-K Chon S-C. Effect of whole body vibration training on mobility in children with cerebral palsy: a randomized controlled experimenter-blinded study. Clin Rehabil. (2013) 27:599–607. 10.1177/0269215512470673 23411791

[53] Lee K Lee S Song C. Whole-Body vibration training improves balance, muscle strength and glycosylated hemoglobin in elderly patients with diabetic neuropathy. Tohoku J Exp Med. (2013) 231:305–14. 10.1620/tjem.231.305 24334483

[54] Liao L-R Ng GYF Jones AYM Huang M-Z Pang MYC. Whole-body vibration intensities in chronic stroke: a randomized controlled trial. Med Sci Sports Exer. (2016) 48:1227–38. 10.1249/MSS.0000000000000909 26918558

[55] Machado A García-López D González-Gallego J Garatachea N. Whole-body vibration training increases muscle strength and mass in older women: a randomized-controlled trial: whole-body vibration in older women. Scan J Med Sci Sports. (2009) 20:200–7. 10.1111/j.1600-0838.2009.00919.x 19422657

[56] Mikhael M Orr R Amsen F Greene D Fiatarone Singh MA. Effect of standing posture during whole body vibration training on muscle morphology and function in older adults: a randomised controlled trial. BMC Geriatr. (2010) 10:74. 10.1186/1471-2318-10-74 20946685 PMC2978213

[57] Pleguezuelos E Pérez ME Guirao L Samitier B Costea M Ortega P Effects of whole body vibration training in patients with severe chronic obstructive pulmonary disease: whole body vibration in COPD. Respirology. (2013) 18:1028–34. 10.1111/resp.12122 23692550

[58] Raimundo AM Gusi N Tomas-Carus P. Fitness efficacy of vibratory exercise compared to walking in postmenopausal women. Eur J Appl Physiol. (2009) 106:741–8. 10.1007/s00421-009-1067-9 19434420

[59] Rees S Murphy A Watsford M. Effects of vibration exercise on muscle performance and mobility in an older population. J Aging Phys Activ. (2007) 15:367–81. 10.1123/japa.15.4.367 18048942

[60] Ruck J Chabot G Rauch F. Vibration treatment in cerebral palsy: A randomized controlled pilot study. J Musculoskelet Neuronal Interact. (2010) 10:77–83. 20190383

[61] Salhi B Malfait TJ Van Maele G Joos G van Meerbeeck JP Derom E. Effects of whole body vibration in patients with COPD. COPD: J Chron Obst Pulm Dis. (2015) 12:525–32. 10.3109/15412555.2015.1008693 26457458

[62] Santin-Medeiros F Rey-López JP Santos-Lozano A Cristi-Montero CS Garatachea Vallejo N. Effects of eight months of whole-body vibration training on the muscle mass and functional capacity of elderly women. J Strength Cond Res. (2015) 29:1863–1869. 10.1519/JSC.0000000000000830 26102257

[63] Simão AP Avelar NC Tossige-Gomes R Neves CD Mendonça VA Miranda AS. Functional performance and inflammatory cytokines after squat exercises and whole-body vibration in elderly individuals with knee osteoarthritis. Arch Phys Med Rehabilit. (2012) 93:1692–700. 10.1016/j.apmr.2012.04.017 22546535

[64] Sitjà-Rabert M Martínez-Zapata MJ Fort Vanmeerhaeghe A Rey Abella F Romero-Rodríguez D Bonfill X. Effects of a whole body vibration (WBV) exercise intervention for institutionalized older people: a randomized, multicentre, parallel, clinical trial. J Am Med Directors Assoc. (2015) 16:125–31. 10.1016/j.jamda.2014.07.018 25282631

[65] Spielmanns M Boeselt T Gloeckl R Klutsch A Fischer H Polanski H. Low-volume whole-body vibration training improves exercise capacity in subjects with mild to severe COPD. Res. Care. (2017) 62:315–23. 10.4187/respcare.05154 27923937

[66] Sucuoglu H Tuzun S Akbaba YA Uludag M Gokpinar HH. Effect of whole-body vibration on balance using posturography and balance tests in postmenopausal women. Am J Phy Med Rehabilit. (2015) 94:499–507. 10.1097/PHM.0000000000000325 26035724

[67] van Nes Ilse JW Latour H Schils F Meijer R van Kuijk A Geurts Alexander C. Long-term effects of 6-week whole-body vibration on balance recovery and activities of daily living in the postacute phase of Stroke. Stroke. (2006) 37:2331–5. 10.1161/01.STR.0000236494.62957.f3 16902175

[68] Wang P Yang L Liu C Wei X Yang X Zhou Y. Effects of whole body vibration exercise associated with quadriceps resistance exercise on functioning and quality of life in patients with knee osteoarthritis: a randomized controlled trial. Clin Rehabilit. (2016) 30:1074–87. 10.1177/0269215515607970 26427960

[69] Wei N Pang MY Ng SS Ng GY. Optimal frequency/time combination of whole body vibration training for developing physical performance of people with sarcopenia: a randomized controlled trial. Clin Rehabilit. (2017) 31:1313–21. 28933611 10.1177/0269215517698835

[70] Rauch F Sievanen H Boonen S Cardinale M Degens H Felsenberg D Reporting whole-body vibration intervention studies: recommendations of the International Society of musculoskeletal and neuronal interactions. J Musculoskelet Neuronal Interact. (2010) 6:193–8. 20811143

[71] Spielmanns M Gloeckl R Gropp JM Nell C Koczulla AR Boeselt T. Whole-Body vibration training during a low frequency outpatient exercise training program in chronic obstructive pulmonary disease patients: a randomized, controlled trial. J Clin Med Res. (2017) 9:396–402. 10.14740/jocmr2763w 28392859 PMC5380172

[72] Choi W Han D Kim J Lee S. Whole-body vibration combined with treadmill training improves walking performance in post-stroke patients: a randomized controlled trial. Med Sci Monitor. (2017) 23:4918–25. 10.12659/MSM.904474 29031023 PMC5652248

[73] Gloeckl R Heinzelmann I Baeuerle S Damm E Schwedhelm A-L Diril M. Effects of whole body vibration in patients with chronic obstructive pulmonary disease – A randomized controlled trial. Respiratory Med. (2012) 106:75–83. 10.1016/j.rmed.2011.10.021 22104540

[74] Park SY Son WM Kwon OS. Effects of whole body vibration training on body composition, skeletal muscle strength, and cardiovascular health. J Exerc Rehabil. (2015) 11:289–95. 10.12965/jer.150254 26730378 PMC4697776

[75] Papa EV Dong X Hassan M. Resistance training for activity limitations in older adults with skeletal muscle function deficits: a systematic review. Clin Interv Aging. (2017) 12:955–61. 10.2147/CIA.S104674 28670114 PMC5479297

[76] Lee PG Jackson EA Richardson CR. Exercise prescriptions in older adults. Am Fam Phys. (2017) 95:425–432. 28409595

[77] Mora JC Valencia WM. Exercise and older adults. Clinics Geriatr Med. (2018) 34:145–62. 10.1016/j.cger.2017.08.007 29129214

[78] Tinetti M. E. (2003). Clinical practice. Preventing falls in elderly persons. N Engl J Med. 348, 42–49. 10.1056/NEJMcp020719 12510042

[79] Curcio F Basile C Liguori I Della-Morte D Gargiulo G Galizia G. Tinetti mobility test is related to muscle mass and strength in non-institutionalized elderly people. Age. (2016) 38:525–33. 10.1007/s11357-016-9935-9 27566307 PMC5266213

[80] Dunn A Marsden DL Nugent E Van Vliet P Spratt NJ Attia J. Protocol variations and six-minute walk test performance in stroke survivors: a systematic review with meta-analysis. Stroke Res Treat. (2015) 2015:484813. 10.1155/2015/484813 25685596 PMC4320847

[81] Bartholdy C Juhl C Christensen R Lund H Zhang W Henriksen M. The role of muscle strengthening in exercise therapy for knee osteoarthritis: a systematic review and meta-regression analysis of randomized trials. Semin Arthritis Rheum. (2017) 47:9–21. 10.1016/j.semarthrit.2017.03.007 28438380

[82] Gojanovic B Henchoz Y Whole-body vibration training: Metabolic cost of synchronous, side-alternating or no vibrations. J Sports Sci. (2012) 30:1397–403. 10.1080/02640414.2012.710756 22845178

[83] Ritzmann R Gollhofer A Kramer A. The influence of vibration type, frequency, body position and additional load on the neuromuscular activity during whole body vibration. Eur J Appl Physiol. (2013) 113:1–11. 10.1007/s00421-012-2402-0 22538279

[84] Chan K-S Liu CW Chen TW Weng MC Huang MH Chen CH. Effects of a single session of whole body vibration on ankle plantarflexion spasticity and gait performance in patients with chronic stroke: a randomized controlled trial. Clin Rehabilit. (2012) 26:1087–95. 10.1177/0269215512446314 23035004

[85] Schuhfried O Mittermaier C Jovanovic T Pieber K Paternostro-Sluga T. Effects of whole-body vibration in patients with multiple sclerosis: a pilot study. Clin Rehabilit. (2005) 19:834–42. 10.1191/0269215505cr919oa 16323382

[86] Silva AT Dias MPF Calixto R Carone AL Martinez BB Silva AM. Acute effects of whole-body vibration on the motor function of patients with stroke: a randomized clinical trial. Am J Phy Med Rehabilit. (2014) 93:310–9. 10.1097/PHM.0000000000000042 24398576

[87] Noyes J Booth A Flemming K Garside R Harden A Lewin S. Cochrane qualitative and implementation methods group guidance paper 3: methods for assessing methodological limitations, data extraction and synthesis, and confidence in synthesized qualitative findings. J Clin Epidemiol. (2018) 97:49–58. 10.1016/j.jclinepi.2017.06.020 29247700

[88] Higgins JP Green S Cochrane Handbook for Systematic Reviews of Interventions. (2019) Available online at: https://handbook-5-1.cochrane.org (accessed March 26, 2019).

[89] Tinetti ME. Performance-oriented assessment of mobility problems in elderly patients. J Am Geriatr Soc. (1986) 34:119–26. 3944402 10.1111/j.1532-5415.1986.tb05480.x

[90] ATS Statement Guidelines for the Six-Minute Walk Test. Am J Respir Crit Care Med. (2002) 166:111–117. 10.1164/ajrccm.166.1.at1102 12091180

